# Amino Acid and Carbohydrate Metabolism Are Coordinated to Maintain Energetic Balance during Drought in Sugarcane

**DOI:** 10.3390/ijms21239124

**Published:** 2020-11-30

**Authors:** Augusto Lima Diniz, Danielle Izilda Rodrigues da Silva, Carolina Gimiliani Lembke, Maximiller Dal-Bianco Lamas Costa, Felipe ten-Caten, Forrest Li, Romel Duarte Vilela, Marcelo Menossi, Doreen Ware, Lauricio Endres, Glaucia Mendes Souza

**Affiliations:** 1 Departamento de Bioquímica, Instituto de Química, Universidade de São Paulo, São Paulo, SP 05508-000, Brazil; augustold@usp.br (A.L.D.); daniizilda080987@gmail.com (D.I.R.d.S.); carolina.lembke@gmail.com (C.G.L.); maximiller@gmail.com (M.D.-B.L.C.); ftencaten@gmail.com (F.t.-C.); 2 Center for Applied Plant Sciences (CAPS), The Ohio State University, Columbus, OH 43210, USA; 3 Escola Superior de Agricultura “Luiz de Queiroz”, Universidade de São Paulo, Piracicaba, SP 13418-900, Brazil; 4 Departamento de Bioquímica e Biologia Molecular, Universidade Federal de Viçosa, Viçosa, MG 36570-900, Brazil; 5 Cold Spring Harbor Laboratory, Cold Spring Harbor, NY 11724, USA; foli@cshl.edu (F.L.); ware@cshl.edu (D.W.); 6 Centro de Ciências Agrárias, Universidade Federal de Alagoas, Rio Largo, AL 57100-000, Brazil; romel@agronomo.eng.br (R.D.V.); lauricioendres@hotmail.com (L.E.); 7 Instituto de Biologia, Universidade Estadual de Campinas, Campinas, SP 13083-862, Brazil; menossi@unicamp.br; 8 USDA ARS NAA Robert W. Holley Center for Agriculture and Health, Ithaca, NY 14853, USA

**Keywords:** plant stress, transcriptome, co-expression network, tricarboxylic acid cycle, TFBS

## Abstract

The ability to expand crop plantations without irrigation is a major goal to increase agriculture sustainability. To achieve this end, we need to understand the mechanisms that govern plant growth responses under drought conditions. In this study, we combined physiological, transcriptomic, and genomic data to provide a comprehensive picture of drought and recovery responses in the leaves and roots of sugarcane. Transcriptomic profiling using oligoarrays and RNA-seq identified 2898 (out of 21,902) and 46,062 (out of 373,869) transcripts as differentially expressed, respectively. Co-expression analysis revealed modules enriched in photosynthesis, small molecule metabolism, alpha-amino acid metabolism, trehalose biosynthesis, serine family amino acid metabolism, and carbohydrate transport. Together, our findings reveal that carbohydrate metabolism is coordinated with the degradation of amino acids to provide carbon skeletons to the tricarboxylic acid cycle. This coordination may help to maintain energetic balance during drought stress adaptation, facilitating recovery after the stress is alleviated. Our results shed light on candidate regulatory elements and pave the way to biotechnology strategies towards the development of drought-tolerant sugarcane plants.

## 1. Introduction

The economic importance of sugarcane goes beyond the production of sugar and biofuels. Interest in studying this crop has increased due to the renewed perception that it can be used for a wide variety of processes, such as the production of polymers through bioprocessing [1]. Brazil plans to expand sugarcane cultivation to pasture areas, a move that satisfies several sustainability criteria but presents challenges because those areas are often affected by stresses such as drought [2,3,4]. Lack of rain can cause losses from 6.3% up to 40% in sugarcane fields, depending on the water shortage [5,6,7], and resulted in extended drought seasons for pasture lands in the center-west and northeast of Brazil [8]. There is a large potential to expand sugarcane production in Latin America, the Caribbean, and Africa, with significant mitigation of greenhouse gas emissions if only 1% of pasturelands were used [9]. But considering future climate changes, it is important that these strategies are accompanied by the development of more resilient crops.

Water deficit affects crop production [10,11] by impeding the plant’s life cycle [12], and requires that the plant undergo rapid, complex, yet coordinated changes in gene expression to ensure its own survival [13]. Drought stress response is a complex system involving optimization of plant growth and survival integrated at several levels [14,15,16,17]. A wide array of hormones, including abscisic acid (ABA), ethylene, and auxin work cooperatively in response to biotic and abiotic stresses, and crosstalk between these pathways has been reported [18,19]. In a previous study, we analyzed the gene expression of sugarcane leaves after 24, 72, and 120 h of water privation [20]. At 24 h, genes related to signal transduction, transporters, DNA metabolism, and protein metabolism were differentially expressed. After 72 h, enrichment in several functional categories reflects modifications of redox, cell wall, and carbohydrate metabolism. After 120 h of stress, RNA and DNA metabolism, which were altered at the early stages of adaptation, return to their original levels. Signal transduction is still altered, possibly to maintain activation of the stress response. At this late stage, various energy pathways are altered (light-harvesting, carbohydrate metabolism, oxidative phosphorylation, and lipid metabolism).

Amino acid and carbohydrate metabolism play important roles in biological cell adaptation [21,22,23]. Carbohydrates are substrates for energy production, osmoregulation, and osmoprotection in drought responses linking growth, development, and carbon status [24]. Amino acids, the building blocks of proteins, play roles in compound biosynthesis, signaling, and stress responses [25]. In plants, the cellular levels and concentration of free amino acids are tightly regulated by metabolic and developmental factors. Their degradation contributes not only to osmotic adjustment, but also to the energy state of the plant, providing a link between carbon and nitrogen metabolism [25]. This connection, which allows plants to cope with suboptimal conditions such as water privation, may be mediated through the tricarboxylic acid (TCA) cycle, the central metabolic cycle of most organisms [17,26].

The links between energetic, amino acid, and sugar metabolism that are coordinated by hormonal changes have not been extensively studied, especially in sugarcane. Until recently, the absence of a reference genome sequence for RNA-seq analysis hampered molecular studies in sugarcane. In the meantime, RNA-seq analysis for sugarcane was performed based on de novo assembly of transcript reads [27,28,29,30,31,32,33,34]. Modern sugarcane cultivars are polyploids, a result of interspecific hybridizations between *S. officinarum* and *S. spontaneum*, as well as other *Saccharum* species and varieties. Due to the sheer genome size (estimated to be 10 G [35,36]), the complex genetic nature of the sugarcane genome, and the release of new sequencing technologies, the first assemblies have been made publicly available only recently: a draft reference genome sequence from the commercial hybrid SP80-3280 [37]; a sequence of a mosaic monoploid from variety R570 [38]; and allele-defined assemblies from the *S. spontaneum* genotype AP85-441 [39], the gene space of *S. spontaneum* [40], and the commercial hybrid SP80-3280 [41]. The latter contains over 373,000 gene models and a wide diversity of homo(eo)logs and regulatory regions.

In this study, we sought to provide a comprehensive view of sugarcane drought responses by analyzing gene expression changes, combining oligoarray and RNA-seq data, using the SP80-3280 gene space [41] as a reference for transcriptome assembly. In addition, we explored the physiological alterations of drought-stressed plants and their manifestations in co-expression networks. Our results suggest that ABA-mediated coordination of carbohydrate metabolism and amino acids degradation provides carbon skeletons to the TCA cycle which plays an important role in maintaining an energetic balance for drought stress adaptation and enables recovery after the stress is alleviated.

## 2. Results

### 2.1. Main Findings of Sugarcane Response to Drought

We performed several drought experiments, in greenhouse and field conditions, in which five sugarcane varieties were subjected to periods without irrigation. The SP90-1638 and RB85-5536 sugarcane varieties cannot be efficiently cultivated in soils with low water retention capacity, and are considered by the Brazilian breeding programs to be sensitive to water stress. Variety RB86-7515 exhibits high agro-industrial productivity (both sugar and biomass), high adaptability, and stability in soils with low natural fertility and soil with low water retention capacity. RB92-579 produces well under water deficit conditions and recovers quickly after stress, and is therefore considered to be tolerant to drought. Variety SP80-3280 is also considered sensitive to water stress, but still ranks among the top 20 sugarcane varieties grown in the state of São Paulo, Brazil, and is being used as a genitor in Brazilian breeding programs. In addition, it is not only a model for large-scale genomic analyses, but also the cultivar with the largest collection of transcriptomics data available [42], and its gene-space assembly includes a large fraction of homo(eo)logs with putative regulatory regions [41].

**Figure 1:**
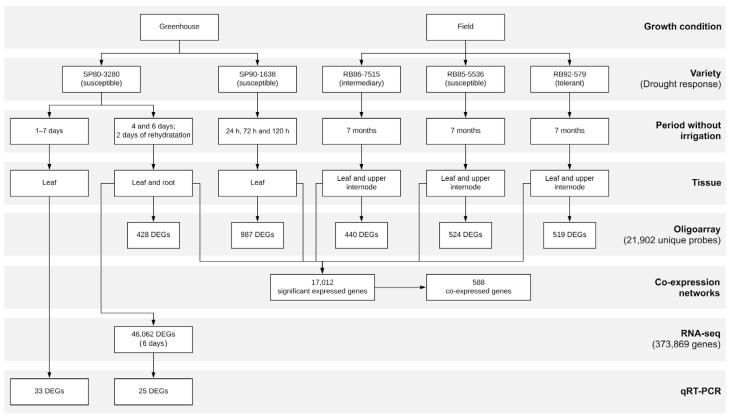
Flowchart showing greenhouse and field experiments with the indicated sugarcane varieties, including time without irrigation, collected tissues, and expression analysis. Gene expression analysis was performed using oligoarray, RNA-seq and/or qRT-PCR, and the number of differentially expressed genes (DEGs) are shown. The 987 DEGs in SP90-1638 were reported previously by Lembke et al. [20]. The respective data sets and the corresponding analyses derived from them are summarized in Table 1.

Samples from leaf, immature internodes, and roots were collected and gene expression was evaluated in oligoarray, RNA-seq, and/or qRT-PCR experiments, as indicated in Figure 1. The main findings with regard to response to drought conditions are summarized in Table 1.

In the oligoarray experiments, we identified 2,898 differentially expressed genes (DEGs) (with overlap among experiments) and 17,012 significantly expressed genes (Figure 1). RNA-seq was conducted only for variety SP80-3280 after a drought experiment in a greenhouse under controlled conditions. Using the SP80-3280 genome reference [41], the average mapping rates of reads from leaf and root samples were 82.35% and 25.83%, respectively. When libraries from both tissues are considered together, a total of 238.8 million reads were mapped onto the SP80-3280 reference genome (Supplementary Table S1). Unaligned reads from root libraries were aligned back to a compendium of 339,873 sequences, including representative genomes of prokaryotes and fungi, as well as all virus genomes available at RefSeq [43]. The alignment rate of the aforementioned unmapped reads was 35.2%, increasing the overall fraction of aligned reads to 53.2%. Bacteria were the leading taxonomic group in both control and drought conditions, representing 97% and 92% of the reads respectively (Supplementary Figure S1). Approximately 60% of the reads obtained under drought conditions correspond to genera *Burkholderia* and *Actinoplanes*. We also detected reads corresponding to the sugarcane yellow leaf virus (ScYLV) and the fungal genus *Fusarium* in drought condition libraries, possibly indicating that variety SP80-3280 is susceptible to these pathogenic species under water deprivation.

### 2.2. Co-Expression Analysis Reveals Four Modules Correlated with Drought and a Potential Role for VQ Proteins in Sugarcane Drought Responses

**Figure 2:**
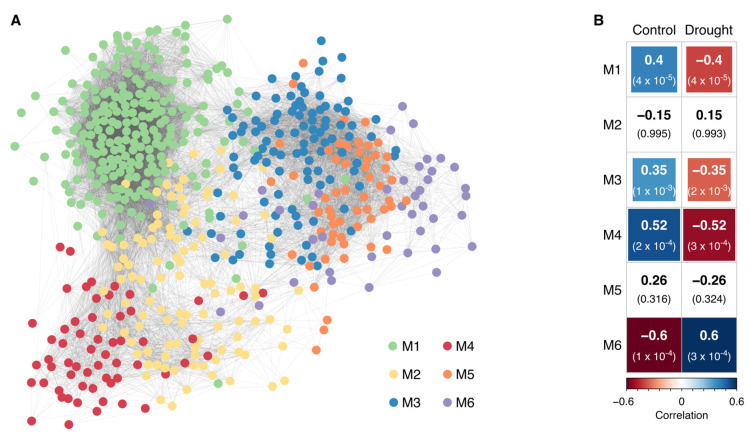
Co-expression analysis of genes from sugarcane under drought stress in both greenhouse and field conditions, classified in six modules. (**A**) Substructures within the co-expression networks. The most enriched GO terms in the six modules are as follows: M1, photosynthesis; M2, small molecule metabolic process; M3, alpha-amino acid metabolic process; M4, trehalose biosynthetic process; M5, serine family amino acid metabolic process; and M6, carbohydrate transport. (**B**) Correlation of the expression levels of co-expressed genes with either drought or control conditions.

To identify a common mechanism underlying the molecular responses of sugarcane to drought conditions, we generated co-expression networks based on expression level above background in all of the oligoarray experiments. A total of 17,012 genes (Supplementary Table S2) that were significantly expressed in the oligoarray experiments were subjected to an unsupervised filtering method based on the inverse gamma distribution from the CEMITool R package [44]. The 5,383 genes that passed the filtering criteria were used, and 588 genes were classified into six modules of 224 (M1), 105 (M2), 102 (M3), 58 (M4), 57 (M5), and 42 (M6) genes (Figure 2A and Supplementary Table S3). Modules M1, M3, M4, and M6 were significantly correlated with drought treatment (Figure 2B); the most enriched GO terms in each module were ‘photosynthesis’ (M1), ‘alpha-amino acid metabolic process’ (M3), ‘trehalose biosynthetic process’ (M4), and ‘carbohydrate transport’ (M6) (Supplementary Table S4).

**Figure 3:**
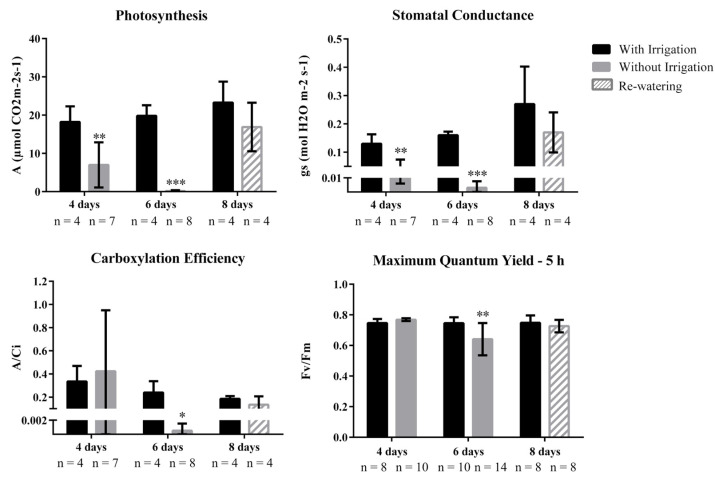
Photosynthesis, stomatal conductance, and carboxylation efficiency are reduced in mild and severe drought stress. Physiological measurements of photosynthesis, stomatal conductance, carboxylation efficiency, and maximum quantum yield in the leaf of sugarcane plants (variety SP80-3280 under greenhouse conditions) submitted to 4 days of water privation, 6 days of water privation, and re-watering for 2 days (8 days). The number of measurements (n) for each physiological trait is shown under each respective bar. Error bars indicate the standard deviation (SD), and asterisks indicate a significant statistical difference, according to t-test, therefore, *; **; *** for *p* ≤ 0.05; *p* ≤ 0.01 and *p* ≤ 0.001, respectively.

Module M1, the major set of co-expressed genes correlated with ‘photosynthesis’, was also enriched for the GO categories ‘photosynthesis light reaction’, ‘plastid organization’, ‘oxidation-reduction process’, and ‘carbon utilization’. After six days of water privation (severe stress), SP80-3280 DEGs identified by oligoarray analysis were enriched in the categories ‘chloroplast relocation’, ‘regulation of stomatal movement’, and ‘water transport’ (Supplementary Figure S2). These results corroborate the physiological parameters of SP80-3280 plants under drought conditions (i.e., after 4 and 6 days of water privation) (Figure 3). The first category (‘chloroplast relocation’) may indicate an attempt of the plant to increase photosynthetic efficiency even at low carbon fixation, and the second (‘regulation of stomatal movement’) reflects physiological observations of near-complete closure of stomata at this time point.

**Figure 4:**
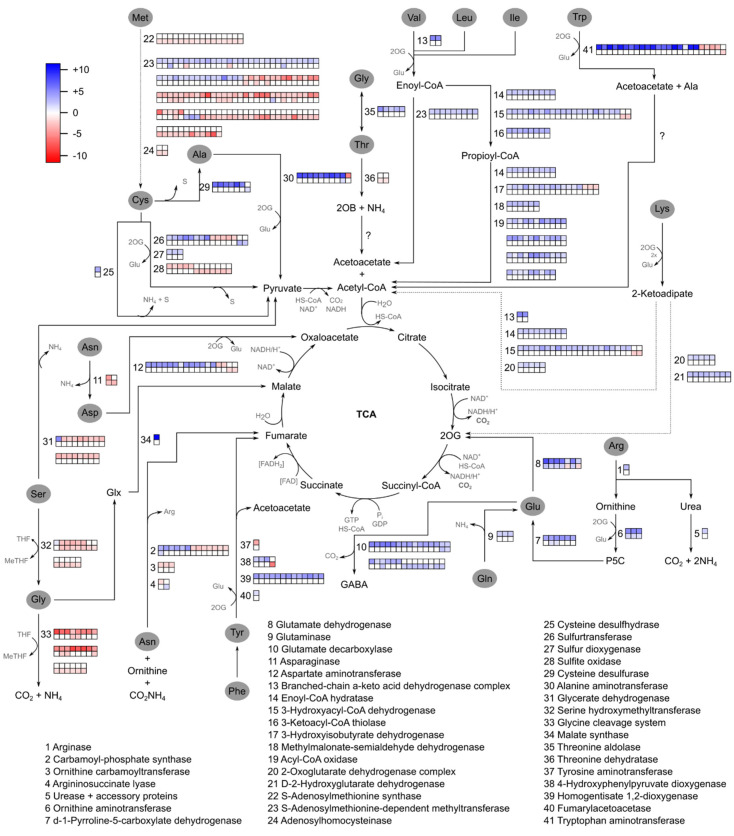
Transcripts involved in the degradation of amino acids are induced, probably to provide carbon skeletons to the TCA cycle. Each square next to an enzyme number represents expression of one transcript at 6 days of drought, as determined by RNA-seq. There are two rows for each enzyme: the top and bottom rows show transcripts differentially expressed in leaves and roots, respectively. Dotted lines need confirmation, according to Hildebrandt et al. [25]. P5C, 1-pyrroline-5-carboxylate; 2OG, 2-oxoglutarate; 2OB: 2-oxobutyrate; 3PG: 3-phosphoglycerate.

Furthermore, oligoarray data from SP80-3280 revealed enrichment of Gene Ontology terms ‘plastid’, ‘thylakoid’, ‘photosynthesis’, and ‘chloroplast’ among genes down-regulated during severe stress (Supplementary Table S5). This reflects the physiological status of the plant, which exhibited a photosynthetic rate of 0.15 µmol CO_2_ m^−2^ s^−1^ under this condition (Figure 3). Transcripts related to the light reaction of photosynthesis were highly down-regulated, as were transcripts related to most of the Calvin cycle. The exceptions to the latter were the pentose phosphate pathway and the biosynthesis of fructose-6-phosphate, which feeds glycolysis (RNA-seq data, Supplementary Figure S3) and consequently, the TCA cycle for energy production. In addition, RNA-seq data showed that transcripts coding for 41 enzymes involved in amino acid degradation were differentially expressed due to drought (Figure 4). The impact of drought on the expression of genes related to carbohydrate and amino acid metabolism in the TCA cycle will be further addressed in the Section 3 (Section 3.3 and Section 3.4, respectively).

Some of the results presented in the flowchart in Figure 1 were expected, e.g., the changes in photosynthesis and stomatal movements, which were observed by measuring the photosynthesis rate and stomatal conductance (Figure 3). A total of 11 genes related to photosynthesis (GO:0015979) appear in the co-expression module M1, and 18 of the co-regulated genes are involved in translation (GO:0006412). Eleven genes with no annotation were also co-regulated in this module, providing insights into their functions.

**Figure 5:**
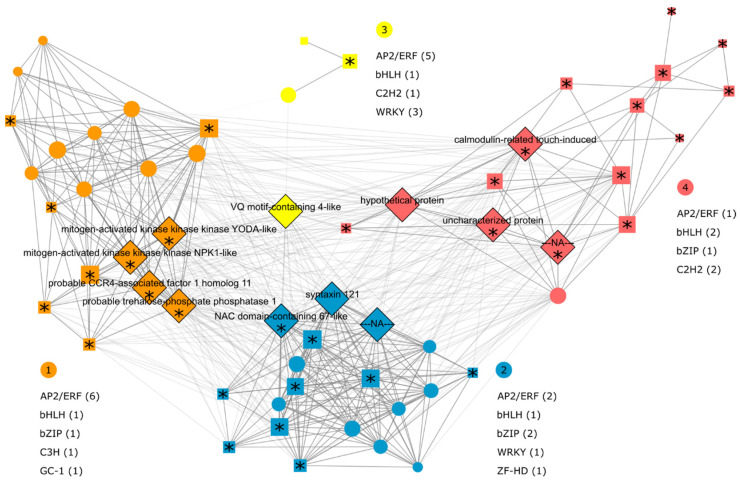
Module M4 from co-expression network of sugarcane submitted to drought is enriched in genes related to hormones and sugars. The transcriptome was analyzed using the CaneRegNet oligoarray platform [20]. Diamonds indicate highly connected genes in the network (top nodes) and asterisks (*) indicate genes differentially expressed between control and treatment (drought) conditions. Numbers in circles (outside the network) represent the clusters of transcripts within the network; listed below the cluster IDs are all de novo predicted transcription factor binding sites (TFBS) in the promoters of the constituent genes, classified based on similarity to annotated motifs deposited in the JASPAR database [46]. The number of predicted TFBS motifs are shown within parenthesis.

In addition to the enrichment of ‘trehalose biosynthetic process’, module M4 also exhibited enrichment of genes related to hormones (Supplementary Table S4). The use of the GLay tool [45] allowed the identification of clusters of highly connected nodes (genes) within the network enabled us to distinguish four clusters within M4. Cluster 1 (orange) includes transcripts responsive to ethylene and auxin; cluster 2 (blue) contains transcripts responsive to ABA; cluster 3 (yellow) contains only four transcripts but with one as top node (i.e., a node with high betweenness); and cluster 4 (red) contains transcripts related to ABA and sugars (Figure 5).

The top nodes in the M4 network include a ‘calmodulin-related touch-induced’ (SCEQLR1029C10.g) and a ‘VQ motif-containing 4-like’ (SCSBLB1036B03.g); the latter is central in the network (Figure 5), but not differentially expressed in any of the oligoarray experiments (Supplementary Table S6). However, the RNA-seq results revealed 32 other VQ motif genes that did not correspond to SCSBLB1036B03.g and were differentially expressed in leaves after 6 days of drought (Supplementary Table S7). In addition, we found the up-regulation of 141 transcripts of WRKY TFs in leaves, versus only 3 (and no VQ motif proteins) in roots. Furthermore, all sugarcane VQ motif proteins induced were predicted in silico (using BaCelLo [47]) to contain nuclear localization signals (Supplementary Table S8). This is interesting because it suggests VQ motif proteins and WRKY TFs are both colocalized and up-regulated in sugarcane under drought stress.

Another top node in the module M4 is ‘probable CCR4-associated factor 1 homolog 11′ (CAF1-11/SCJFRT1059F04.g/cluster 1), a component of the CCR4-NOT complex involved in mRNA deadenylation and RNA-induced gene silencing; the gene is induced by drought and ABA12. Other ABA-regulated or -related genes in M4 include ‘probable phosphatase 2C’ (PP2C/SCCCCL3005D01.b/SCVPRZ2039D09.g), ‘ABA responsive element binding factor 1′ (SCCCLR1C03C05.g), and ‘abscisic acid 8-hydrolase 1′ (SCRFRZ3058E03.b).

We next combined expression data with genomic sequence data to study the regulatory regions of the co-expressed genes. The availability of the SP80-3280 gene space sequence [41] allowed us to study the regulatory regions of the genes of interest. Of 58 co-expressed genes or SAS (Sugarcane Assembly Sequences [42]) in M4, 50 were similar to 181 genes in the SP80-3280 gene-space assembly, ranging from one to 11 gene copies per transcript (Supplementary Table S9). We identified 181 transcription factor binding sites (TFBSs) in M4, of which 90 did not exhibit similarity to the motifs in the JASPAR database [47]; however, these may represent regulatory sequences not yet characterized, and are therefore candidates for future analysis. The remaining 32 are from eight different classes: 13 AP2/ERF, 5 bHLH, 4 bZIP, 4 WRKY, 3 C2H2 zinc finger factor, 1 C3H zinc finger, 1 homeodomain ZF-HD, and 1 GC-1.

### 2.3. De Novo Predicted TFBSs within Co-Expressed Genes’ Regulatory Regions Are Enriched among Drought-Responsive Genes

To identify putative TF-coding transcripts, we mapped the RNA-seq transcriptome assembly onto the transcriptomes of other reference species. To identify putative orthologs via sequence similarity, predicted protein sequences in sugarcane were aligned to those in *Sorghum bicolor* and *Arabidopsis thaliana*. A total of 237,052 transcripts in the sugarcane assembly mapped directly to 27,357 protein-coding genes in sorghum, and 150,255 sugarcane transcripts mapped to 15,674 protein-coding genes in Arabidopsis (Supplementary Table S10). To observe regulatory regions, we further limited this gene space to transcripts with a 1,500-bp upstream promoter region in this assembly, yielding a search space of 107,077 transcripts. Canonical representative motifs from 11 TF families were taken from previous DNA affinity purification sequencing (DAP-seq) experiments in *Arabidopsis thaliana* [48] and the promoter regions of all orthologs were scanned for putative TFBSs using FIMO [49].

The selected TF families include: (i) TFs families that were de novo predicted to be enriched among the M4 gene co-expression network, including WRKY, bHLH, AP2/ERF, C2H2, C3H, ZFHD, and bZIP; (ii) TFs families not enriched in the M4 gene co-expression network, however previously reported as responsive to drought, such as NAC [50], MYB [51], and Trihelix [52]; and (iii) the Dof family, which is reported to be mainly involved in the control of flowering time [53].

When looking at the genome-wide search space, target genes that were differentially expressed in drought were significantly enriched for TFBS hits by AP2-ERF, WRKY, bHLH, and C2H2 family TFs, as predicted by the M4 co-expression network. In comparison with other families not enriched in the network, we found that MYB and Trihelix target genes, previously reported to be involved in crosstalk in abiotic stresses [51,52], were enriched for TFBS hits in DEGs under drought conditions. In addition, the primarily developmental NAC and Dof families were depleted for TFBS, and the lower-ranking TF families of C3H, ZFHD, and bZIP in the M4 network were not significantly enriched. The enrichment of TFBSs further suggests some level of interaction between the WRKY, AP2/ERF, C2H2, and bHLH families and drought-related abiotic stresses (Supplementary Table S11).

### 2.4. Auxin, Ethylene, and ABA May Coordinate the Drought Response of Sugarcane

To continue our analysis, we performed daily evaluations of the expression of genes related to the hormones auxin, ethylene, and ABA, categories that were enriched in the M4 module. SP80-3280 plants were subjected to drought for 7 days; impaired photosynthesis became evident on the third day, with the lowest rate occurring on the sixth day of stress (Supplementary Figure S4A). In addition, stomatal conductance, transpiration, and soil humidity were also affected after the third day of water privation (Supplementary Figure S4B–D, respectively).

Expression of the auxin response factor (SCCCCL3120A10.b), which controls expression of auxin response genes, was up-regulated after 6 days without irrigation. The expression of the auxin-responsive gene SAUR11 (SCCCRT1C01F08.g), involved in auxin signaling, was repressed before withholding of water and was up-regulated on the fifth and sixth days without irrigation. By contrast, ABP5 (SCCCRT2001C12.g) was repressed on the fifth day, and the expression of the auxin-responsive gene IAA7 (SCCCRZ2003H05.g), which represses the early auxin response, was down-regulated at that time point (Supplementary Figure S5).

Regarding to ethylene, we observed induction of the genes encoding ‘ACC synthase’ (ACS-SCJLRT1006C03.g) and ‘ACC oxidase’ (SCEZLR1009E06.g), both of which are involved in the biosynthesis of ethylene, after the fourth day of stress (Supplementary Figure S6). We observed induction of ‘ethylene-insensitive 2′ (SCSBHR1056H08.g), which acts downstream of the ethylene receptors, on the fifth, sixth, and seventh day without irrigation (Supplementary Figure S6). Despite that, we observed no significant induction of ‘ethylene-responsive factor 1′ (SCCCCL4002B07.g) or ‘ethylene response sensor 2′ (SCSBAD1086C06.g) (Supplementary Figure S6). The up-regulation of ACS and ‘ACC oxidase’ may be explained by the down-regulation of ‘S-adenosylmethionine (SAM) synthase’ (RNA-seq data, Supplementary Table S7) (SAM is a precursor of aminocyclopropane-1-carboxylic acid which is a precursor of ethylene) in roots, and the lack of a change in expression in leaves may reflect a strategy to more efficiently use scarce substrate for ethylene biosynthesis. This is consistent with the overall repression of enzymes involved in methionine and cysteine catabolism (RNA-seq data). However, the ethylene that may be synthesized appears insufficient to elicit the expression of ethylene-responsive genes.

The expression of genes related to ABA exhibited different patterns in leaves and roots. Expression of many genes related to ABA pathways increased in leaves after 4 and 7 days of drought, although expression of some genes decreased (Supplementary Figure S7). On the third and fourth days of drought, SCRLLR1038F07.g was induced in leaves (Supplementary Table S12). After 6 days of drought, transcripts involved in ABA-dependent regulation were identified in the oligoarray and RNA-seq data. Examples include ABRE, AUX/IAA, DREB, EREBP/AP2, and NAC transcription factors (TFs), type 2C protein phosphatases (PP2C), and SnRK2 kinases (Supplementary Table S12). In addition, the transcripts from the ABA biosynthesis pathway were repressed in leaves (Supplementary Figure S8) but induced in roots. ‘Zeaxanthin epoxidase’, which catalyzes the first step in the ABA biosynthesis pathway, was repressed in leaves and induced in roots. Transcripts encoding the enzymes ‘9-cis-epoxycarotenoid dioxygenase’ and ‘abscisic-aldehyde oxidase’ were up-regulated in roots and down-regulated in leaves (Supplementary Figure S8).

### 2.5. Different Transporters Participate in the Drought Responses of Sugarcane

In the M6 co-expression network (Supplementary Figure S9), we observed the following top nodes: ‘chloride channel CLC-c-like’ (SCVPLB1015F06.g–induced in roots after 4 days of drought), ‘cold-regulated 413 plasma membrane 2′ (SCUTLR2015A11.g), and ‘aquaporin’ (SCAGLR2033E03.g). Other transporters were also identified as members of the M6 co-expression network: ‘transmembrane amino acid transporter family’ (SCSBRZ3118B10.g—induced in roots after 6 days of drought), ‘organic cation carnitine transporter 7′ (SCCCLR1067A05.g), and ‘amino acid transporter’ (SCEQRT1030B05.g). This observation corroborates the amino acid mobilization pathways indicated in Figure 4.

We also observed repression of glucose import and water and ion transport transcripts (Supplementary Table S7) in roots, possibly reflecting a strategy for increasing water resistance to avoid the loss of water to the environment under conditions associated with low rates of transpiration or periods of water shortage [54,55]. Our data revealed a large decrease in transpiration at 6 days of stress (Supplementary Figures S4 and S11), and the increase in hydraulic resistance may be a response of drought-stressed pot-cultivated sugarcane plants, as the organism’s search for water is space-limited. The biological process ‘water transport’ is represented mainly by the down-regulation of aquaporins, such as aquaporins TIP2-3 (SCBGRT1052E01.g), TIP2-1 (SCJFRT1010C08.g), and PIP2-6 (SCEQRT1024B11.g). Down-regulated aquaporin transcripts were also found by Mirzaei et al. [56] and may point to a response or adaptation of the plant aimed at quickly changing membrane permeability [55]. Our data reveal a direct relationship between an increase in hydraulic resistance and low rates of transpiration [54] at 6 days of drought, transpiration was 0.30 mmol H_2_O m^−2^ s^−1^, and water use was 0.45 A/E (instantaneous) and 6 A/gS (intrinsic) (Supplementary Figure S10).

### 2.6. Genes Related to Cell Wall, Cold Stress, and Lipid Metabolism Are Induced in Leaves and Repressed in Roots during Severe Stress

One functional category that yielded different results between leaves and roots was chromatin remodeling. In response to biotic and abiotic stress, chromatin remodeling is a powerful and flexible tool for making rapid changes in gene expression, leading to adaptation [57,58,59]. Our data show that histones, ‘histone deacetylase’, ‘lysine-specific histone demethylase’, and ‘histone-lysine N-methyltransferase’ were in most cases up-regulated in leaves (Supplementary Table S7). By contrast, no histones or histone-related transcripts were up-regulated in roots. These results suggest greater remodeling of activated genes in leaves than in roots.

Despite the lower rate of RNA-seq read mapping in roots, a similar number of DEG transcripts related to abiotic stress were identified in leaves and roots. The difference was that cold stress-related transcripts were induced in leaves and repressed in roots, and a similar pattern was observed for ‘Heat’, ‘Drought/Salt’, and ‘Misc.’ (Supplementary Figure S11).

The fold changes of up- and down-regulated transcripts related to the cell cycle and cell division, DNA repair, and response to oxidative stress were similar between leaves and roots, but a higher percentage of repressed transcripts was observed in roots (Supplementary Figure S11). This might reflect impairment of root growth in terms of both cell division and cell expansion in stressed sugarcane plants cultivated in pots with limited space. Reduction in root volume and root dry mass after water suppression in drought-stressed sugarcane greenhouse cultivated plants has previously been reported [60,61]. An overall reduction in the expression of genes related to cell wall metabolism was observed in roots, whereas genes from this category were generally induced in leaves (Supplementary Figure S11). Seventy-eight expansins were repressed in roots and none in leaves, whereas 19 members of this class were induced in leaves vs. only one in roots (Supplementary Table S7). Genes associated with lipid metabolism were also induced in leaves and repressed in roots after 6 days of drought (Supplementary Figure S11).

### 2.7. Significantly Expressed Transcripts after Re-Watering Reflect Repression of Drought Responses and Induction of Plant Recovery

Next, we analyzed overrepresented transcript classes after re-watering. To this end, we selected significantly expressed transcripts with a coefficient of variation >120 among all samples and conditions. The initial group of 13,216 transcripts was further selected, and the group used for the clustering analysis consisted of 4539 transcripts. Among a total of 22 clusters, two exhibited different behavior after rehydration: Cluster 4 included transcripts that were induced after re-watering (Supplementary Figure S12A), whereas Cluster 5 consisted of transcripts that were repressed (Supplementary Figure S12B). Up-regulated biological processes included ‘Cell Division’, ‘Cycle and Growth’, ‘Ribosome Biogenesis’, and ‘Translation’, which relate to the ability of the plant to recover its basic functions and restore growth and development capacity, as well as ‘Threonine Metabolic Process’ and ‘Valine Catabolic Process’. In addition, we found a higher percentage of transcripts related to Cell Cycle and Cell Division repressed in roots, during re-watering after drought (Supplementary Figure S13).

By contrast, Cluster 5 was enriched in genes associated with ‘Reductive TCA Cycle’, ‘Threonine Biosynthetic Process’, and ‘Cellular Amino Acid Metabolic Process’, as well as ‘Response to Abscisic Acid’ and ‘Glutathione Metabolic Process’. The latter two processes suggested that neither ABA nor ROS responses were necessary anymore, as the stress had ended.

Moreover, there was an enrichment of transcripts associated with ‘Plastid Cellular Component’ and ‘Ribosomal Large Subunit Binding Molecular Function’ (Supplementary Table S5). The former indicates recovery of the photosynthetic apparatus, as demonstrated by the recovery of photosynthesis (Figure 4), whereas the latter demonstrates recovery of protein synthesis after the stressful condition is removed. Up-regulation of heat shock proteins was also observed in both leaves and roots (Supplementary Tables S6 and S7). These factors are important for the recovery process because they are involved in protecting the cell from injuries such as those caused by drought stress.

## 3. Discussion

### 3.1. Sugarcane Response to Drought

As previously mentioned, we have performed several drought experiments, in greenhouse and field conditions, using five sugarcane varieties subjected to distinct periods without irrigation (Figure 1). In addition, gene expression of sugarcane different tissues was evaluated using oligoarray, RNA-seq, and/or qRT-PCR experiments.

Before further discussing the impact of drought on sugarcane gene expression, it is important to highlight that a large fraction of RNA-seq reads did not map to the sugarcane genome reference [41]. Amog unmapped reads, we found an enrichment of bacteria of phylum Actinobacteria, which includes genus *Actinoplanes*, is a common response of the root rhizosphere to drought stress [62,63], possibly due to the ability of these bacteria to access metabolites released by the plant under these conditions [64]. Similarly, endophytic colonization of *Burkholderia* species increases drought stress resilience of maize, wheat, and other plants under drought conditions [65,66,67]. In addition, these diazotrophic bacteria also promote growth in sugarcane [68,69,70].

With regards to the sugarcane response to drought at the gene expression level, and as summarized in Table 1, we saw that this stress largely impacts plant’s photosynthetic ability and primary metabolism, including amino acid and carbohydrate metabolism. In addition, co-expression and differential expression analysis highlights candidates phytohormones and TFs as key regulatory elements regulating sugarcane response to drought.

For instance, we found a ‘VQ motif-containing 4-like’ (SCSBLB1036B03.g) as a top node in a co-expression network enriched for carbohydrate metabolism. It has been reported that VQ proteins are important co-regulators of plant defense responses and play crucial roles in abiotic stress responses [71,72], seed development, and photomorphogenesis [73]. They have a conserved single amino acid motif and may be central in the network because they regulate many developmental processes [71,72,73]. The importance of VQ proteins in the response to drought conditions has already been reported for grasses. For instance, among 61 VQ motif members in maize, 41 are differentially expressed during drought [72]. In addition, VQ, WRKY transcription factors, and MAP kinases form a closely related complex to fine-tune gene transcription [73]. Perruc et al. [74] demonstrated that VQ15 interacts with calmodulin (CAM) and responds to osmotic stress in a calcium-dependent manner. VQ motifs and WRKYs engage in complex interactions that can either stimulate or repress the WRKY DNA-binding activity [73]. Furthermore, WRKY TFs may regulate their own expression through feedback mechanisms, as well as VQ motif protein expression [75,76]. Thus, the results corroborate the hypothesis that VQ proteins interact with several WRKY TFs [77].

The distribution of de novo predicted TF classes among co-expressed genes in M4 clusters (Figure 5) indicates AP2/ERF as the most frequent. This class of TFBS is characterized by motifs that allow the binding of transcription factors responsive to ethylene, a hormone involved in the senescence process and the drought response [78]. However, we also identified WRKY TFBS motifs as enriched among co-expressed genes in M4. This class of motifs engages in complex interactions with VQ motif-containing proteins and can stimulate or repress binding of the TF to DNA [73]. *Oryza sativa* VQ domain proteins are differentially regulated following ABA or drought treatment, suggesting they may be subject to the same sort of regulation by ABA [71]. In *Vitis vinifera*, 72.2% of the promoter regions of VQ genes contain an ABRE element, and may therefore be regulated by ABA [77]. In addition, some VQ proteins are induced by ethylene, suggesting that they are also regulated by this hormone [77]. The central position of this transcript in the sugarcane co-expression network, along with transcripts related to ABA, ethylene, and auxin corroborates the results from a previous study [71] and suggests that in sugarcane this transcriptional regulator, through its interaction with WRKY TFs, may be involved in the crosstalk between different hormone pathways in response to abiotic stress. Interestingly, the WRKY family plays an important role in the drought response of *Sorghum bicolor* [79].

### 3.2. Genes from ABA Pathways Are Expressed in Different Patterns in Leaves and Roots under Drought Conditions

The phytohormone ABA plays a role in perceiving and responding adaptively to drought stress. At the same time, it acts synergistically with other phytohormones to better adapt plants to various stresses. Expression of many genes related to ABA pathways increased in leaves after 4 and 7 days of drought, although expression of some genes decreased (Supplementary Figure S7). On the third and fourth days of drought, SCRLLR1038F07.g was induced in leaves (Supplementary Table S12). The hypothetical protein encoded by this gene contains an Interpro domain (IPR007650-PFAM/PTHR33059-PANTHER/PTHR33059:SF7-PANTHER) involved in ABA responses, and was previously reported to confer drought resistance in wheat [80].

As previously mentioned, transcripts involved in ABA-dependent regulation were identified in the oligoarray (6 days) and RNA-seq data analysis. As examples, we highlight ABRE, AUX/IAA, DREB, EREBP/AP2, and NAC transcription factors (TFs), type 2C protein phosphatases (PP2C), and SnRK2 kinases (Supplementary Table S12). Furthermore, ABA biosynthesis pathway transcripts, such as ‘Zeaxanthin epoxidase’, which catalyzes the first step in the ABA biosynthesis pathway, were predominantly down-regulated in leaves (Supplementary Figure S8) and up-regulated in roots. On the other hand, transcripts encoding the enzymes ‘9-cis-epoxycarotenoid dioxygenase’ and ‘abscisic-aldehyde oxidase’ were up-regulated in roots and down-regulated in leaves (Supplementary Figure S8). The first step in ABA catabolism, which inactivates ABA signaling, is the formation of 8′-hydroxy ABA by ‘ABA 8′ hydroxylase’. Transcription of the gene encoding this enzyme was repressed in leaves and induced in roots. Thus, both biosynthetic and catabolic enzymes were induced in roots. Moreover, recent work showed that phaseic acid, a product of ABA catabolism formed by the isomerization of 8′-hydroxy ABA, has some ABA-like effect (reviewed by Rodriguez [81]).

### 3.3. Sugar Metabolism is Channeled to Input Energy and Carbon into the TCA Cycle

The enriched GO categories related to sugar metabolism in the M4 module of the co-expression network are as follows: ‘oligosaccharide Biosynthetic Process’, ‘disaccharide Biosynthetic Process’, ‘trehalose biosynthetic process’ and ‘trehalose metabolic process’ (Supplementary Table S4). Enrichment of trehalose biosynthetic process, along with the predominant induction of phosphoenolpyruvate carboxylase (PEPC) transcripts in leaves, indicates that as in other species, sugarcane may have a mechanism for simultaneous activation of PEPC and ‘nitrate reductase’ (NR) triggered by an increase in ‘trehalose 6-phosphate’ (Tre6P) [24]. During the day, Tre6P influences the partitioning of photo-assimilates between sucrose, organic acids, and amino acids via posttranslational regulation of PEPC and NR in leaves. Induction of Tre6P elevates amino acid levels by stimulating nitrate assimilation, indirectly leading to an increase in the levels of TCA cycle intermediates [24].

The importance of Tre6P in sugarcane leaves, as well as ‘trehalose-phosphate phosphatase 1′ (TPP/SCEPLR1051A07.g) and ‘trehalose-phosphate synthase 6′ (TPS/SCCCCL3002E04.b), may be related to their osmoregulatory functions [24]. As indicated by its co-expression with ABA related transcripts, TPP may alter stomatal conductance [82,83]; moreover, *AtTPPG* and *AtTRE1* gene promoters contain motifs that implicate MYB and WRKY TFs in regulation by ABA. The co-expression in module M4 of TPP with VQ motif proteins, along with the known interaction of VQ motif proteins with WRKY TFs, suggests a role for these proteins in regulating trehalose metabolism in an ABA-dependent pathway. This relationship between VQ motif genes and Tre6P has not been described in other plants.

In sugarcane, the raffinose and stachyose family of oligosaccharides (RFOs) may be involved in osmoprotection and in preventing oxidative damage, as in other species [84,85]. In leaves and roots at 6 days of drought, we observed down-regulation of transcripts involved with the galactose catabolic process and up-regulation of transcripts involved with RFO biosynthesis and raffinose transport (oligoarray and RNA-seq data, Supplementary Table S7). A ‘stachyose synthase precursor’ (SCJFLR1017E09.g) was up-regulated in leaves after 4 days of drought and in roots after 6 days (Supplementary Table S6).

Other DEGs involved with sugar metabolism were ‘soluble starch synthase II-2′ (SCSGSB1009B08.b), down-regulated in leaves after 6 days of drought (Supplementary Figure S14), as well as ‘UDP-glucose 6-dehydrogenase’ (UGDH/SCQGLR1019G02.g) and ‘sucrose synthase 4′ (SuSy4/SCEPCL6023F02.g) (Supplementary Figure S14); the one last was the top node of module 6 (Supplementary Figure S9).

Finally, the up-regulation of ‘sulfur dioxygenase’ (cysteine metabolism) (Supplementary Table S6) in leaves after 6 days of drought may be induced by carbohydrate starvation [25] and corroborates the association between carbohydrate metabolism and amino acid catabolism.

### 3.4. Amino Acid Catabolism Provides Carbon Skeletons for the TCA Cycle and Compensates for Repression of Photosynthesis and Inhibition of the Calvin Cycle

Together, the physiological data presented above demonstrate a significant decrease in photosynthesis, stomatal conductance, carboxylation efficiency (Figure 3), and effective quantum efficiency after 6 days of water privation (Supplementary Figure S10). Limitation of stomatal conductance may be linked with the induction of ‘cysteine desulfhydrase’ (Figure 4), which alters stomatal opening [86,87] and may reflect a strategy for recovering the photosynthetic apparatus. This enzyme is involved in the synthesis of iron-sulfur clusters and is probably relevant for electron transfer chains [25]. The decrease of physiological parameters indicate damage in photosystem II, leading to a reduction in carbon fixation due to low CO_2_ influx, limiting the Calvin cycle and causing not only damage to the photochemical apparatus but also carbon starvation [25,88,89,90,91].

During starvation in plants, carbon-nitrogen balance must be coordinated, and ‘glutamate dehydrogenase’ (converts L-glutamate into alpha-ketoglutarate) and ‘glutamate decarboxylase’ (converts glutamate to oxoglutarate and GABA, respectively) are involved in amino acid oxidation and degradation [25,92,93,94] to meet energy demands during stress. We observed a prominent induction of glutamate dehydrogenase and glutamate decarboxylase in leaves of SP80-3280 after 6 days of drought (Figure 4). Nitrogen mobilization to aid ion homeostasis may also be occurring in stressed roots, as evidenced by induction of an asparaginase enzyme (Figure 4).

Biological functions related to amino acid metabolism were enriched in five out of six co-expression modules: M1, M2, M3, M5, and M6. In addition, amino acid metabolism is altered in response to drought stress in other plants [95,96]. After 6 days of drought, we observed induction of ‘nitrate reductase’, ‘nitrite reductase’, and ‘glutamine synthetase’, which are involved in assimilation of nitrogen into amino acids (Supplementary Table S6). Expression of transcripts encoding ‘aspartate–ammonia ligase’, involved in asparagine biosynthesis, was also induced on day 6 (Supplementary Table S6). Asparagine, along with glutamine and arginine, plays a key role in nitrogen storage and transport [97], and the induction of transcripts encoding ‘asparaginase’ (Figure 4) indicating that nitrogen mobilization might contribute to ion homeostasis in stressed roots.

We also observed induction of most amino acid catabolism related transcripts that provide intermediates for the TCA cycle (Figure 4); this induction was especially prominent in leaves. The higher induction of tryptophan catabolism may be due to the fact that its degradation produces acetoacetate, a high-energy compound [98], and alanine, which can be further catabolized to yield pyruvate and generate energy. The induction of tryptophan catabolism under water privation conditions has been observed as well as the role of this amino acid in increasing the biosynthesis of different phytohormones, such as ABA and auxin, in addition to phytoalexins and other metabolites [99].

By contrast, amino acid catabolism enzymes that do not provide precursors for the TCA cycle (i.e., glycine and serine) and those involved in the catabolism of amino acids for which it would be necessary to execute more steps to synthesize the precursors of TCA cycle, were predominantly down-regulated; for example, this was the case for methionine (Figure 4).

According to Asbahi et al. [100], there is a strong relationship between ABA content and amino acid accumulation in response to drought, and this is one of the most important defense mechanisms against drought in many plants. Furthermore, according to Nambara et al. [101], it is likely that ABA-mediated closure of stomata is necessary for the initiation of the plant’s slow response to dehydration, such as changes in free amino acid levels. This relationship has been confirmed in the literature [100,101,102]. As shown in Figure 3, we observed significant decreases in stomatal conductance even during moderate stress, and this may be associated with induction enzymes involved in Lys, Thr, and Gln catabolism (Figure 4). Therefore, there may be an occurrence of the synthesis of amino acids induced by ABA, which are quickly degraded to provide precursors for the TCA cycle.

The enzyme ‘Δ-1-pyrroline-5-carboxylate synthetase’ (P5CS1/SCJFLR1073H12.g), which is involved in proline biosynthesis, an important component of the osmotic stress response [12,92,103], was altered after 4 days of stress in roots and after 2 days in leaves (Supplementary Table S13). P5CS also generates NADP^+^, which may be recycled in the pentose phosphate pathway, thereby maintaining the activity of the TCA and Calvin cycles and decreasing ROS production during moderate stress. Consistent with this, at 2 days of drought, physiological data indicates increased stomatal closure (Supplementary Figure S4) but no alteration in the Calvin cycle.

## 4. Materials and Methods

### 4.1. Plant Material and Experimental Conditions

Previous work from our group identified a small number of genes regulated after 24 h of water privation [20] (GEO series record GSE33574). In this work we performed a drought progression expression experiment to detect the best time points for transcriptome analysis based on induction of genes from hormone-related pathways. For the drought progression experiment, one-eye set of sugarcane were planted in 20-L pots containing 2:2:1 soil:substrate:vermiculite, arranged in a completely randomized design, and cultivated for 7 months in a greenhouse in Prof. Erich Grotewold’s Laboratory at The Ohio State University (Columbus, OH, USA). Before water withholding, pots were irrigated until the water was drained away (moisture near Field Capacity (FC)). In each day of the experiment, soil humidity was measured at a depth of 10 cm using the soil moisture meter MO750 (EXTECH Instruments, Nashua, NH, USA). Irrigation was ceased for 7 days and physiological measurements were taken. Water was supplied on the 8th day of the experiment. For RNA extraction, the third leaf from top to bottom of the stalk with clearly visible dewlap (leaf +3) was collected each day over 7 days of water privation.

For the drought experiment (samples harvested after 4 or 6 days of stress and rehydration), two one-eye sets of sugarcane were planted in 20-L pots containing soil, arranged in a completely randomized design, and cultivated for 5 months before stress in a greenhouse at Prof. Laurício Endres’s Laboratory at the Federal University of Alagoas (Maceio, Brazil). Before water withholding in half of the pots, water content was maintained near FC. Three daily soil humidity measurements were taken in each pot at a depth of 5 cm using the humidity sensor model SM200 (DELTA-T Devices, Cambridge, England). Leaf (L+1) and root samples were collected after 4 or 6 days of water privation, and followed by 2 days of re-watering (8 days). After collection, all samples were immediately frozen in liquid nitrogen and kept in dry ice until storage at −80 °C.

In addition to the greenhouse experiments, we have used the expression data of samples from leaf +1 and upper internodes from 3 sugarcane varieties: RB86-7515 (intermediary tolerant to drought), RB92-579 (tolerant to drought), and RB85-5536 (lower drought tolerance) from RIDESA (Rede Interuniversitária para o Desenvolvimento do Setor Sucroenergético). Plants were field-grown in Campo Alegre, Alagoas, Brazil (9°45′32″ S, 36°13′09″ W), and samples were collected 7 months after planting under irrigation or without irrigation (rainfed) and firstly used for studying the microtranscriptome of field-grown sugarcane plants [6].

### 4.2. Physiological Analysis

In the drought progression experiment, gas exchange measurements at leaf L+1were performed using a LICOR LI-6400 Portable Photosynthesis System (LI-COR, Lincoln, NE, USA). All measurements were performed between 9:00 and 11:00 a.m. The plants were allowed to recover for 24 h (between day 7 and day 9) after they were watered again. Consequently, no physiological measurements were taken on day 8.

In the 4- and 6-day water privation experiment, leaf water potential was measured at leaf L+2 using a Scholander Pressure Chamber (Soil Moisture, Equipment Corporation, Santa Barbara, CA, USA) at 4:30 a.m. A portable infrared gas analyzer (IRGA, ADC Bioscientific, Hoddesdon, UK) and a light source of 1.123 μmol m^−2^ s^−1^ were used to measure photosynthesis rate (A), stomatal conductance (gs), and transpiration (E) in the time interval between 8:00 and 11:00 a.m. at leaf L+1. Using the above variables, carboxylation efficiency (A/ci) and intrinsic water use efficiency (A/gs) were calculated. A modulated fluorometer 051-FL(OPTI-SCIENCES, Hudson, NH, USA) was used for maximum quantum efficiency measurement of PSII (Fv/Fm) at predawn and at midday with saturating light pulses of 1 s. Before the measurement was taken, leaves were adapted to the dark for 20 min. The same leaf was used to measure effective quantum yield (φPSII) between 10:00 a.m. and 12:00 p.m. as described in Maxwell; Johnson [89].

### 4.3. RNA Extraction

Total RNA was extracted from leaves and roots with modifications from Zeng and Yang protocol [104]. Then, 100–300 mg of frozen shredded tissue was mixed with 900 µL prewarmed (65 °C) extraction buffer [2% CTAB, 4% PVP, 10 mM Tris-HCl (pH 8), 25 mM EDTA, 2 M NaCl, 4 mL DEPC (diethylpyrocarbonate) Milli-Q Water, 2% β-mercaptoethanol]. Samples were incubated at 65 °C for 15 min and with vigorous shaking every 5 min. After incubation, an equal volume of CIA (1 isoamyl alcohol: 24 chloroform) was added and the samples were centrifuged at 20,000× *g* for 10 min. The supernatant was re-extracted four times with the same volume of CIA. The supernatant was mixed with 0.25 vol of 10 M LiCl and maintained at 4 °C overnight. Samples were centrifuged at 16,000× *g* for 40 min at 4 °C and the pellet was washed twice with 75% ethanol. Precipitated RNA was air-dried for 10 min and re-suspended in 30–50 μL of DEPC treated water. RNA concentration was determined using a NanoDrop spectrophotometer (Thermo Fisher Scientific, Waltham, MA, USA). RNA samples were treated with DNase I, amplification grade (Invitrogen, Carlsbad, CA, USA), and cleaned using a RNeasy Mini Kit (Qiagen, Hilden, Germany). RNA integrity was assayed using the Agilent RNA 6000 Pico Kit (Agilent Technologies, Santa Clara, CA, USA) on a 2100 Bioanalyzer (Agilent Technologies, Santa Clara, CA, USA).

### 4.4. Gene Expression

#### 4.4.1. Oligoarrays

Oligoarray sample preparation and analysis was performed as described in Lembke et al. [20], using two biological replicates for control and treated samples from: (i) 4 or 6 days of water privation, and 2 days of re-watering (greenhouse-grown plants); and (ii) 7 months after planting (field-grown plants). Only RNA samples with RIN ≥ 6.5 were selected, and hybridizations were performed using the customized sugarcane Agilent oligoarray platform [20]. The arrays contained probes to detect the sense and antisense expression of 14,522 different Sugarcane Assembled Sequences (SAS), which are reference sequences for sugarcane transcripts [42]. Hybridized oligoarray slides were scanned in GenePix 4000B scanner (Molecular Devices, San Jose, CA, USA), and image data were extracted with the aid of the Feature Extraction 9.5.3 (Agilent Technologies, Santa Clara, CA, USA) using the two-color oligoarray referential in the Agilent platform intensity [105] from Cy3 and Cy5 was corrected and normalized using the Lowess function [106] implemented in the R software. Microarray data files are deposited at the Gene Expression Omnibus (GEO) public database, series record GSE125069.

#### 4.4.2. RNA-Seq

RNA-Seq analysis was performed using three biological replicates for control and treated root and leaf samples after 6 days of water privation (greenhouse-grown plants). This time point was chosen because it presented the highest number of differentially expressed transcripts in oligoarray experiments. As cited above, RNA integrity was assayed using the Agilent RNA 6000 Pico Kit (Agilent Technologies, Santa Clara, CA, USA) on a 2100 Bioanalyzer (Agilent Technologies, Santa Clara, CA, USA) and only samples with RNA Integrity Number above 6.5 were used for library preparation using the TruSeq Low Input Library Prep Kit (Illumina, San Diego, CA, USA, catalogue number FC-134-2002) at the Beijing Genomics Institute. One μg of RNA was used for library preparation and sequencing was performed on an Illumina Sequencing Machine HiSeq4000 (PE150) and a total of 6G of data were generated for each sample. RNA-seq data files are deposited at the Sequence Read Archive (SRA), accession number PRJNA628529.

#### 4.4.3. Real-Time Quantitative Reverse Transcription PCR (qRT-PCR)

For qRT-PCR analysis, cDNA was synthesized using SuperScript (Invitrogen, Carlsbad, CA, USA) with oligo(dT)s, using 500 ng of total RNA for each sample. Three biological replicates and three technical replicates were used for each time point validation (nine replicates in total). Primers were designed based on SAS sequences using the Primer Express 2.0 (Applied Biosystems, Foster City, CA, USA) software, and only primers with an amplification efficiency between 90 and 110% were used (Supplementary Table S14). Reaction mixtures consisted of 5 μL of 1.5 μM primer mix, 1 μL of cDNA (diluted 1:10) and 6 μL of Fast SYBR Green Master Mix (Applied Biosystems, Foster City, CA, USA). Reactions were performed on 7500 Fast Real-Time PCR System (Applied Biosystems, Foster City, CA, USA). For primer SCSGLR1045D05.g, primer concentration was reduced to 312.5 nM. Expression ratio was estimated using REST 2009 (Relative Expression Software Tool, Qiagen, Hilden, Germany) as described in [107]. The endogenous genes for each tissue were identified using the geNorm software [108] (Supplementary Table S15).

### 4.5. Data Analysis

#### 4.5.1. Detection of Differentially Expressed Genes

Oligoarray data analyses were performed using the SUCEST-FUN (http://sucest-fun.org/wsapp/) tools and web environment. Differentially expressed genes (DEGs) were defined by the HT-self method [109], adapted to the Agilent oligonucleotide oligoarray platform [20]. Differentially expressed SAS were re-annotated by a search of six-frame translated SAS sequences against the NCBI non-redundant protein database using BLASTP (e-value < 1 × 10^−5^). Next, automated functional annotation was performed using the Blast2GO framework [110]. Finally, SAS search against Clusters of Orthologous Groups (COGs) allowed the categorization of transcripts in specific functional categories.

RNA-Seq reads mapping to the sugarcane SP80-3280 gene space assembly was done using Bowtie2 [40]. Gene coverage was estimated using HTSeq [110] and DEGs were identified with R package DESeq2 [111], combining technical replicates and applying a *p*-value cut-off of 0.01 after adjustment by the FDR procedure [112]. For both oligoarray and RNA-Seq, DEGs were annotated using Blast2GO and characterized by Gene Ontology terms (biological process, molecular function, and cellular component). For blastx an E-value was set to 1x10^-5^ and the number of blast hits set to 10. DEGs were used to perform functional classifications and enrichment analysis using AgriGO [113,114], ReVIGO [115], and MapMan [116].

#### 4.5.2. Analysis of Unaligned Reads from Root Libraries

To identify the proportion of reads from root libraries not mapped to the SP80-3280 genome, which could represent transcripts from contaminant microorganisms, we aligned those reads to a RefSeq compendium of representative genomes of prokaryotes, fungi, and viruses, totaling 339,873 sequences, 42,109, using bwa-mem [117] with default parameters. Genera abundance and richness from contaminant reads was accessed using the MetaPhlAn (Metagenomic Phylogenetic Analysis) software with default parameters [118].

#### 4.5.3. Co-Expression Analysis

For the co-expression analysis, we considered the oligoarray expression data generated in this study in addition to two experiments performed previously by our group. The experiment with variety SP90-1638 was carried out under greenhouse conditions and leaf samples were collected after 24, 72, and 120 h of water withholding. Leaf samples from respective controls were also collected for gene expression analysis [41]. The experiment with varieties RB86-7515, RB85-5536, and RB92-579 was carried out under field conditions without irrigation in a dry season [6]. Leaves and internode I from 7-month-old plants were sampled for transcriptomic analysis. For oligoarrays replication control, two biological replicates and dye swaps were carried out. Data processing; normalization and analysis were performed according to Lembke et al. [20].

Log2-transformed expression data were used for co-expression modules prediction using the CEMiTool R package [44], using default parameters except that apply_vst = TRUE and diss_thresh = 0.9. The adjacency matrix was calculated by estimating the Pearson’s correlation coefficient between all pairs of genes and raised to a soft thresholding power (β) of 14. Gene Set Enrichment Analyses, implemented in the CEMiTool R package, reveals which modules are correlated with either Drought or Control samples. Fisher’s exact test, implemented in the ‘TopGO’ R package [119], was used for Gene Ontology enrichment analysis of each module. Node and edge files were generated for use with the Cytoscape network visualization program [120]. Finally, the community clustering algorithm (GLay), implemented in the Cytoscape tool clusterMaker [45], was used to detect substructures within the co-expression networks.

#### 4.5.4. Transcription Factor Binding Site (TFBS) Prediction within Regulatory Regions of Co-Expressed Genes

Co-expressed transcripts (SAS) were used to identify gene sequences from the SP80-3280 assembly via sequence alignment with the ‘GMAP’ tool [121], with -n 200 and default parameters. For TFBS prediction in regulatory regions, a 1,500-bp sequence upstream of the gene start codon was selected, excluding sequences with overlapping coding regions. We used a stochastic method implemented through the MotifSuite tool [122] to identify and analyze TFBSs. The background model was constructed using the CreateBackgroundModel tool, considering all intergenic regions of the SP80-3280 assembly [41].

This analysis was conducted separately for genes corresponding to each of the four co-expression clusters within the M4 module, with the goal of identifying transcription factors specific to each cluster. The identification of enriched motifs was performed using the MotifSampler tool, searching for up to six motifs per sequence with up to three replicates and sizes of 6, 8, 10, and 12 nucleotides. The motifs obtained from multiple tool runs were sorted based on their scores and grouped according to the similarity of their PWMs using the MotifRanking tool in order to decrease redundancy in the final data set. Motifs identified 10 or more times in a total of 100 runs were considered to be valid for subsequent analyzes.

The valid motifs were compared to 489 annotated plant motifs available in the JASPAR database [47] using the MotifComparison tool with Kullback–Leibler distance (KL) methods with default parameters and p-BLiC with parameters -n 40 and n−1 overlap in relation to the size of the analyzed motif. In addition, we also used TOMTOM [123] with E-value threshold < 0.01. The set of valid motifs was also mapped against itself to search for redundancies between the clusters and sizes of motifs analyzed. All valid motifs were mapped to the sequences of the promoter regions using the MotifLocator tool with parameter -t 0.9, as well as to a set of 10,000 randomly selected gene promoter regions, in order to calculate the mean alignment rate of each motif in the genome. Motifs were considered enriched in the analyzed network when present at >50% of the mapping rate in the set of random sequences.

#### 4.5.5. TFBS Enrichment Analysis

A summary workflow for the TFBS enrichment analysis is presented in Supplementary Figure S15. Predicted protein sequences annotated in the sugarcane RNA-seq transcriptome assembly were aligned to genome-wide peptide databases in *A. thaliana* (TAIR10) and *S. bicolor* (NCBIv3) using BLASTP (e-value < 0.001). The top BLAST results (top five in Arabidopsis and top 10 in sorghum) were filtered for similarity and query coverage. A higher confidence sugarcane/Arabidopsis ortholog list was obtained by combining sugarcane/sorghum orthologs with known sorghum/Arabidopsis orthologous relationships in Gramene [124].

To generate a list of sugarcane genes with valid regulatory regions, all gene model transcripts in the high-confidence sugarcane/Arabidopsis ortholog list were filtered for those that contained a 1500-bp promoter region upstream of the transcription start site in this transcriptome assembly (Supplementary Table S10).

To confirm the presence of binding sites for transcription factors of interest, we performed a scan of canonical Arabidopsis TF binding motifs for genes with valid regulatory regions. Clustering of differentially expressed genes in the M4 network (Figure 5) was enriched for transcription factor families of interest under drought conditions. We obtained five representative canonical motif position weight matrices from Arabidopsis DAP-seq experiments [48] for each of the WRKY, bHLH, AP2/ERF, bZIP, C2H2, C3H, ZFHD, bHLH, MYB, Dof, and NAC transcription factor families. In parallel, we found all sugarcane genes annotated as a transcription factor (Supplementary Table S16), using the web tool ‘Predicion’ from the Plant Transcription Factor Database (PlantTFDB v5.0-http://planttfdb.cbi.pku.edu.cn/) [125]. Finally, for each of the 11 TF families, we subset the sugarcane corresponding genes, and then identified their corresponding orthologs in the sugarcane/Arabidopsis mapping. We then mapped each Arabidopsis TF ortholog’s targets using DAP-seq interaction data [48], if available, back to sugarcane transcript IDs, resulting in a list of putative sugarcane targets for each TF family of interest. We then used the motif scanning software FIMO [49] with default parameters to locate putative TFBSs in the 1,500-bp promoter regions for all genes in each list. We also used a novel Python script to generate “consensus” binding sites where multiple transcription factors are predicted to bind at the same coordinates of a promoter region. Genes with more than one TFBS binding event or a “consensus” TFBS in the promoter region were marked as such. Enrichment analysis of DEGs with a TFBS was performed for each TF of interest by hypergeometric test using the SciPy [126] package in Python. To account for multiple testing, a Bonferroni correction was performed with a critical value of 4.55 × 10^−3^.

## 5. Conclusions

In this study, we conducted a transcriptome analysis of sugarcane in response to drought stress and re-watering, using several approaches. Five different sugarcane cultivars were studied, and the analysis was complemented with an in-depth analysis of cultivar SP80-3280 genomic sequences, making it possible to map the RNA-seq reads and identify TFBSs. The expression analysis was accompanied by physiological observations of plants subjected to drought. Expression of 46,062 genes was altered.

In general, the differentially regulated transcripts were involved in photosynthesis, hormone, sugar and amino acid metabolism, chromatin remodeling, cell cycle, cell division, and cell wall (Table 1). We also detected a large number of transcripts that have no BLAST matches or that are derived from unknown or hypothetical genes. Future studies focused on characterizing such sequences may reveal that they are long non-coding RNAs (lncRNAs), undescribed genes, or sugarcane-specific transcripts representing new sources of variability; all possibilities represent potential future targets for transformation and plant breeding studies.

Our findings show that despite global alteration of sugarcane metabolism due to drought stress, major changes in carbon fixation occur in leaves, which we speculate is a response to ABA synthesized in roots. In addition, amino acid metabolism seems to play a fundamental role in sugarcane drought response mechanisms: in part by affecting signaling and osmoregulation, but especially by maintaining energetic balance by providing carbon skeletons to the TCA cycle, thereby aiding in plant survival during the stress and providing conditions for recovery after stress ceases. Together, these mechanisms serve to maintain water use efficiency and the energy status of the plant despite low carbon gains due to reductions in photosynthesis and stomatal conductance. Finally, our results provide an overview of the molecular physiological pathways underlying sugarcane drought responses, paving the way toward the development of drought-tolerant sugarcane plants.

## Figures and Tables

**Table 1:** Summary of alterations identified in sugarcane under drought.

Altered State	Evidence	Analysis	Figure/Table
Photosynthesis	Overrepresentation among the DEGs of genes related to chloroplast relocation, regulation of stomatal movement, and water transport	ReViGo Networks (oligoarray)	Supplementary Figure S2
	Enrichment among down-regulated genes of Gene Ontology terms related to plastid, thylakoid, photosynthesis, and chloroplast	Enrichment (oligoarray)	Supplementary Table S5
	Down-regulation of genes involved in light reactions of photosynthesis and most of the Calvin Cycle	DEGs (RNA-seq)	Supplementary Figure S3
	Reductions in the photosynthesis rate and stomatal conductance	Physiology measurements	Figure 3
Hormones	Co-expression of genes related to ethylene, auxin, and ABA	Co-expression (oligoarray)	Figure 2
	Identification of hormone-related transcription factor binding sites	Identification of TFBS in co-expression genes	Figure 2
	ABA-related DEGs	Drought progression (qRT-PCR)	Supplementary Figure S7
	Ethylene-related DEGs	Drought progression (qRT-PCR)	Supplementary Figure S6
VQ protein as important regulation of drought responses	Top node in a co-expression network (M4) and 32 differentially expressed VQ protein-coding genes after 6 days of drought in leaves	Co-expression analysis (oligoarray) RNA-seq	Figure 2 Supplementary Table S7
Sugar Metabolism	Induction of PEPC, Tre6P in leaves Up-regulation of RFO biosynthesis Reduction of galactose catabolism Induction of sucrose synthesis	DEGs and MapMan (RNA-seq)	Supplementary Tables S6 and S7 Supplementary Figure S14
Amino acid to TCA	Amino acid–related categories in various modules of co-expression	Co-expression analysis	Figure 2 Supplementary Table S4
	Induction of enzymes involved in nitrogen assimilation	DEGs (RNA-seq)	Supplementary Table S7
	Induction of enzymes involved in amino acid catabolism	DEGs (RNA-seq)	Figure 3
Chromatin remodeling	Induction of histone-related genes	DEGs (RNA-seq)	Supplementary Table S7
Cell cycle, Cell Division	A higher percentage of repressed transcripts in roots	DEGs (RNA-seq)	Supplementary Figure S13
Cell Wall	Induction of expression in leaves and repressed expression in roots	MapMan (RNA-seq)	Supplementary Figure S11

## Supplementary Materials

Supplementary materials can be found at https://www.mdpi.com/1422-0067/21/23/9124/s1.
