# Supplementary material for: Amino Acid and Carbohydrate Metabolism Are Coordinated to Maintain Energetic Balance during Drought in Sugarcane
Source: Int J Mol Sci. 2020 Nov 30;21(23):9124. doi: 10.3390/ijms21239124 (PMC7729667; doi:10.3390/ijms21239124)
Supplement: Supplementary file 1 [file ijms-21-09124-s001.zip › Diniz_Supp_files_MDPI/Diniz_Supplementary_figures.docx]

**Supplementary Figures**

**Amino acid and carbohydrate metabolism are coordinated to maintain energetic balance during drought in sugarcane**

Augusto Lima Diniz^1†^, Danielle Izilda Rodrigues da Silva^1,2,3†^, Carolina Gimiliani Lembke^1^, Maximiller Dal-Bianco Lamas Costa^1,4^, Felipe ten-Caten^1^, Forrest Li^5^, Romel Duarte Vilela^6^, Marcelo Menossi^7^, Doreen Ware^5,8^, Lauricio Endres^6^ & Glaucia Mendes Souza^1*^

^1^ Departamento de Bioquímica, Instituto de Química, Universidade de São Paulo, São Paulo, Brazil

^2^ The Ohio State University, Columbus, OH, USA

^3^ Escola Superior de Agricultura “Luiz de Queiroz”, Universidade de São Paulo, São Paulo, Brazil

^4^ Departamento de Bioquímica e Biologia Molecular, Universidade Federal de Viçosa, Minas Gerais, Brazil

^5^ Cold Spring Harbor Laboratory, New York, NY, USA

^6^ Centro de Ciências Agrárias, Universidade Federal de Alagoas, Alagoas, Brazil

^7^ Instituto de Biologia, Universidade Estadual de Campinas, Campinas, Brazil

^8^ United States Department of Agriculture, USA

^†^These authors contributed equally to this work

^*^ Corresponding author: glmsouza@iq.usp.br

*
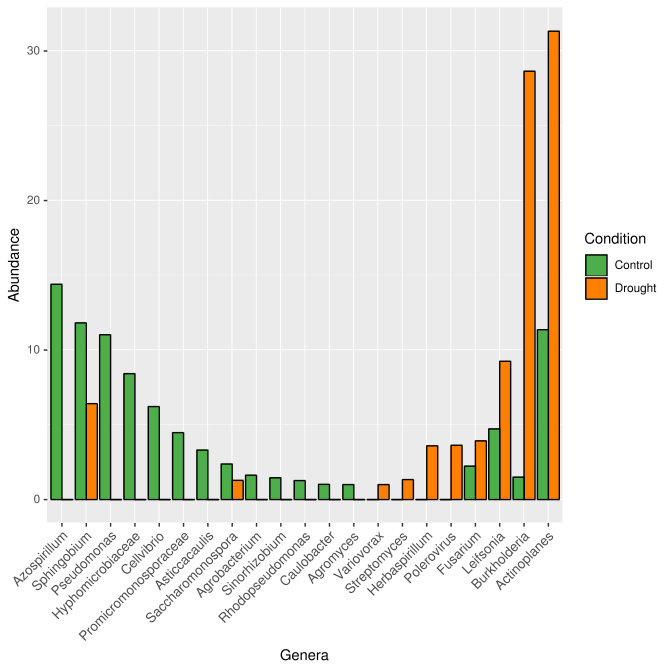
***Supplementary Figure 1 – Most abundant genera (> 1%) identified in control and drought libraries.**


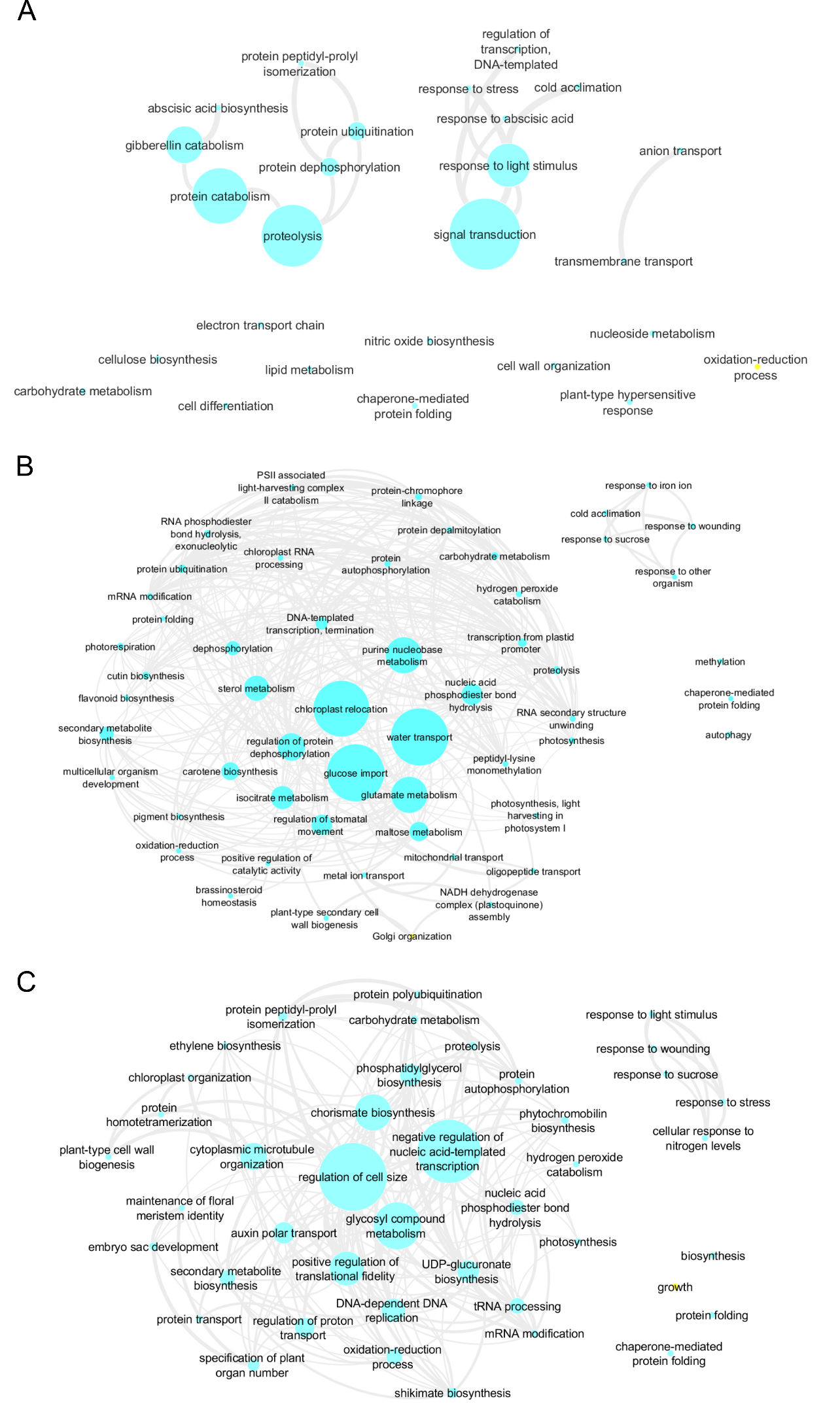


**Supplementary Figure 2 – ReViGo Networks summarizing differentially expressed transcripts in leaves, as detected by oligoarray, after 4 days (A), 6 days (B), and re-watering (C).**


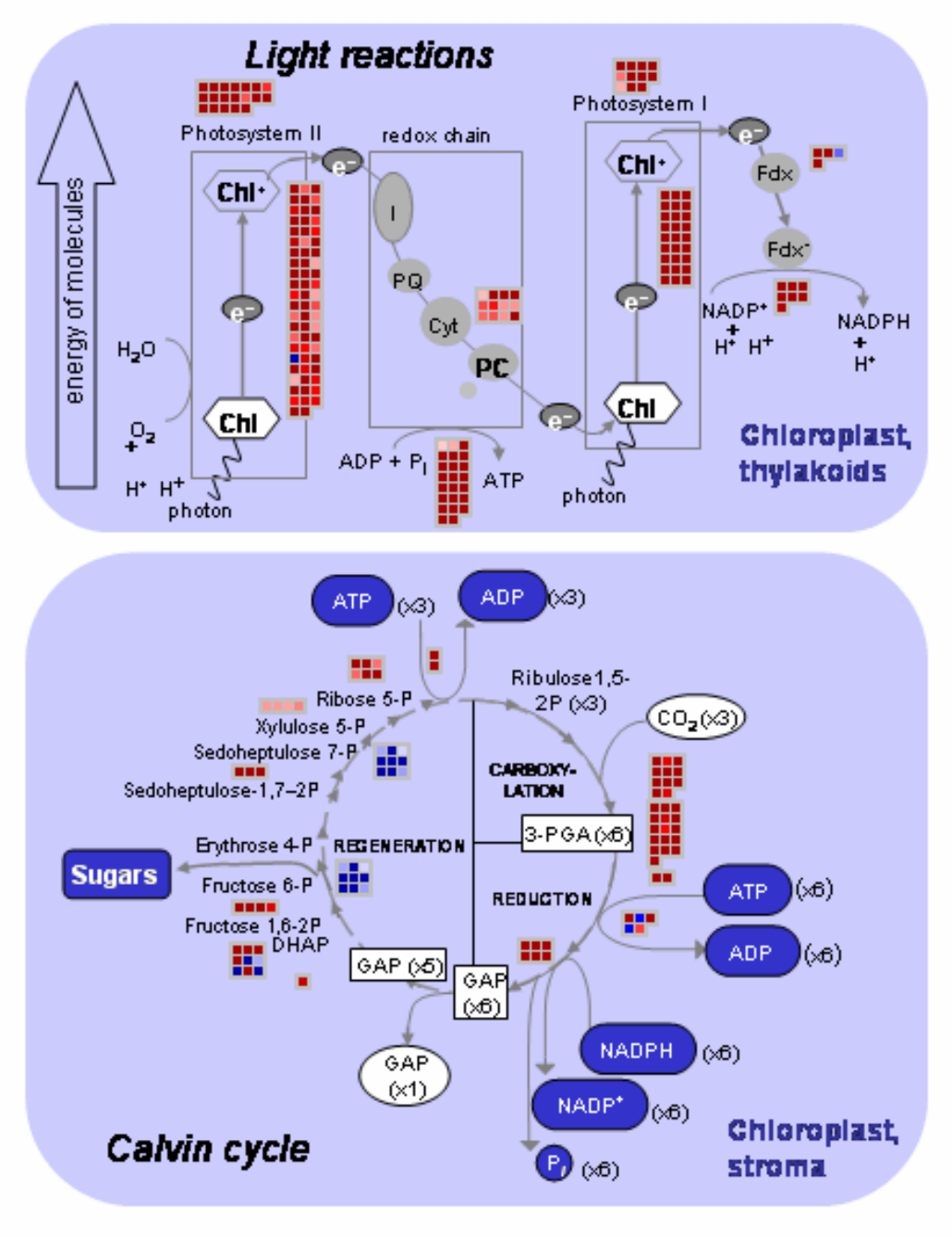


**Supplementary Figure 3 – Expression of transcripts from the photosynthesis and Calvin cycle are repressed**. Transcripts from the pentose phosphate pathway are induced. Mapman figures show repressed (red squares) and induced transcripts (blue squares) in RNA-seq data from leaves subjected to drought for 6 days.

1


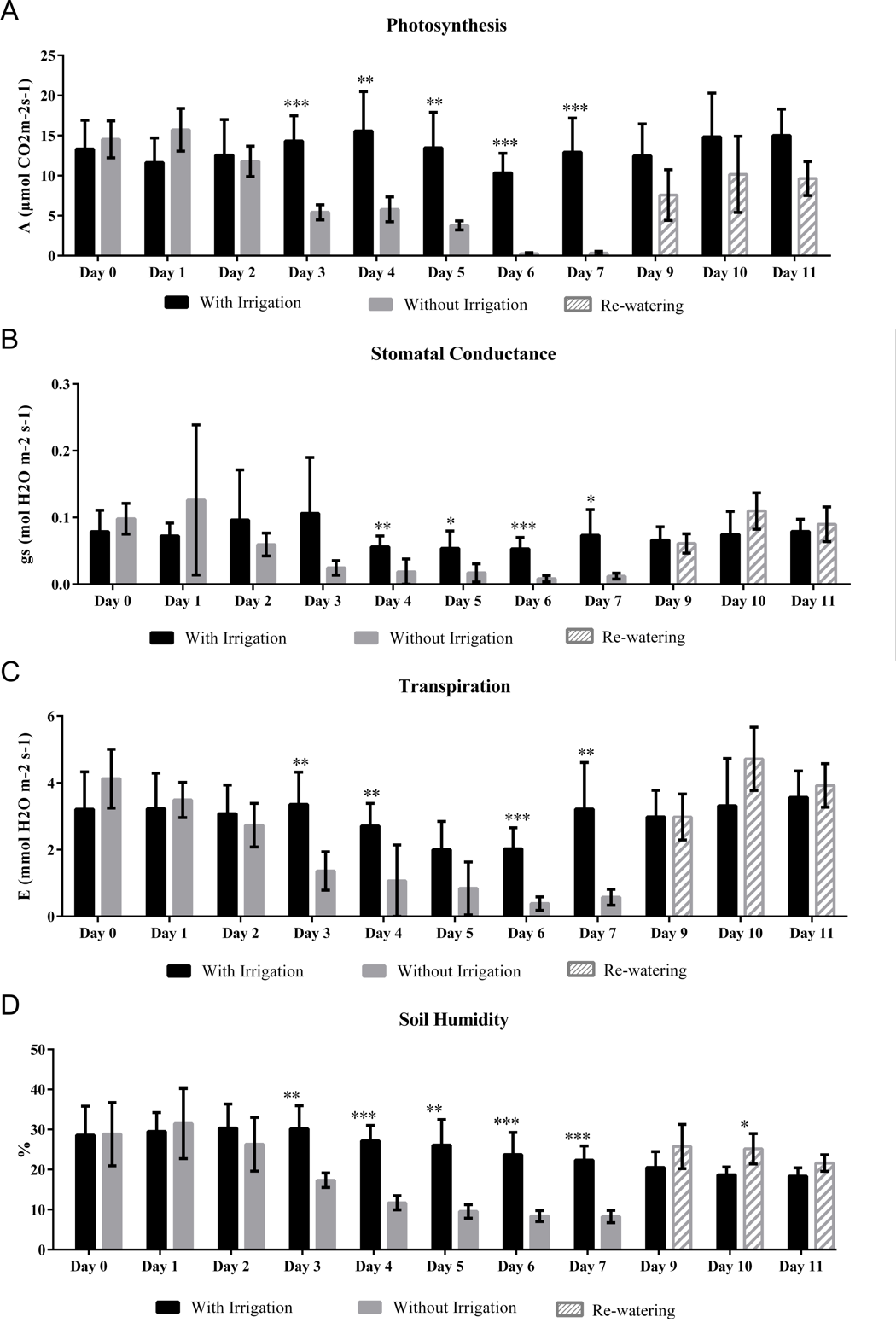


**Supplementary Figure 4 – Graphs of (A) photosynthesis, (B) stomatal conductance, (C) transpiration, and (D) soil Humidity during the 7-day drought experiment and after re-watering (day 9-11).** Water was supplied on the 8th day of the experiment. Drought stress was extreme after 6 days, and the plants fully recovered 24 hours after re-watering. Bars show standard deviation, and asterisks indicate significant statistical differences (t test); *; **; *** indicate p≤0.05; p≤0.01, and p≤0.001, respectively.


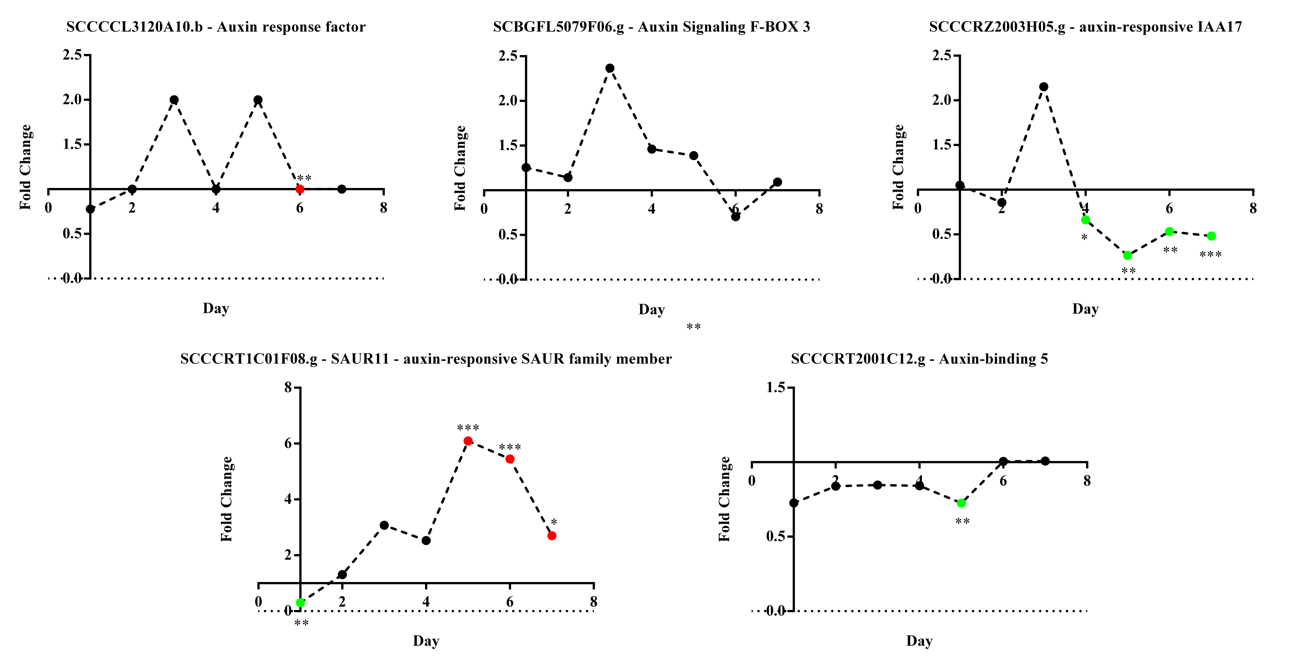


**Supplementary Figure 5 - Expression of genes related to auxin is modulated after 3 days of drought.**


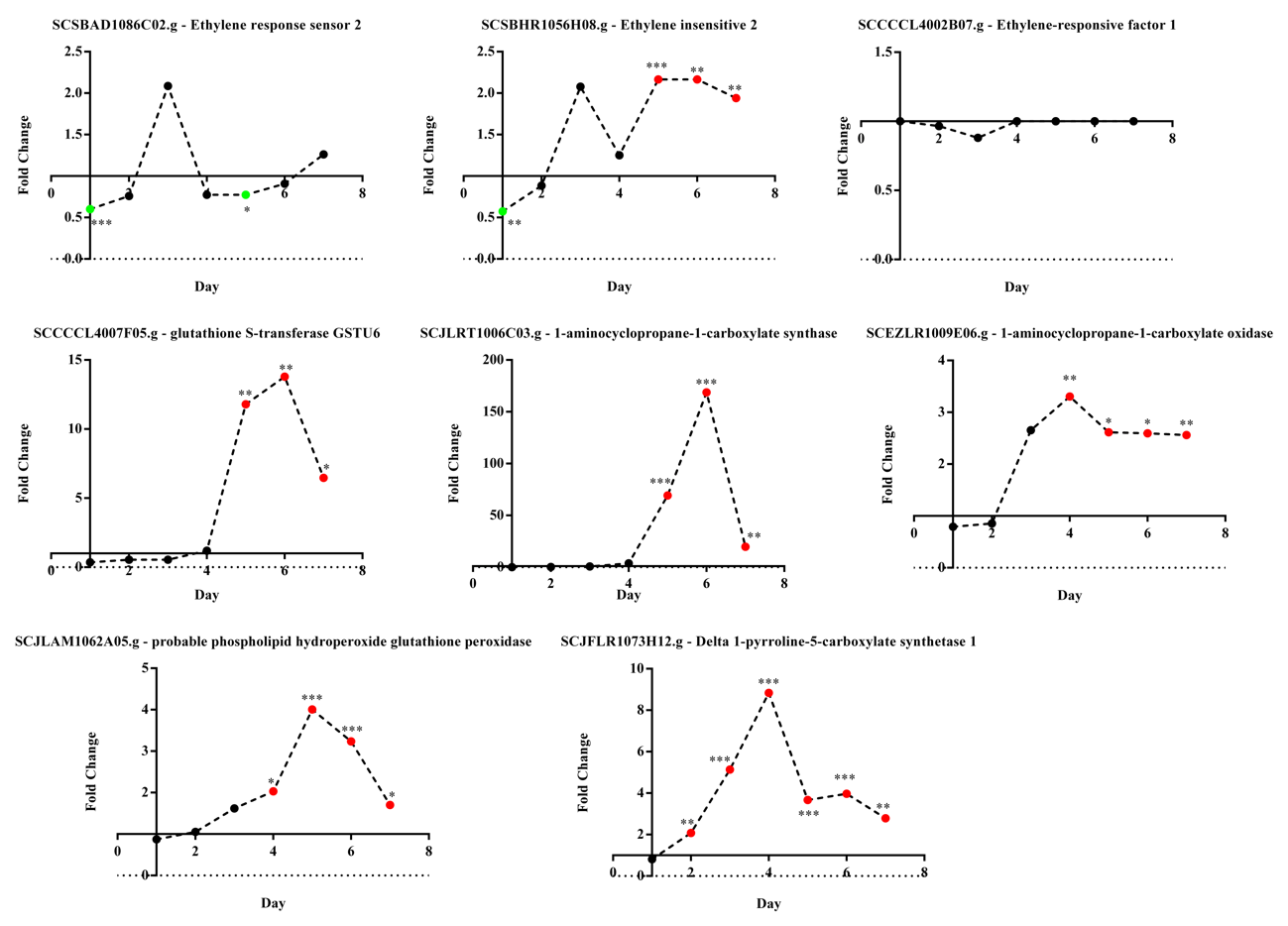


**Supplementary Figure 6 – Expression of genes related to ethylene under drought conditions, measured daily, for 7 days without irrigation.**


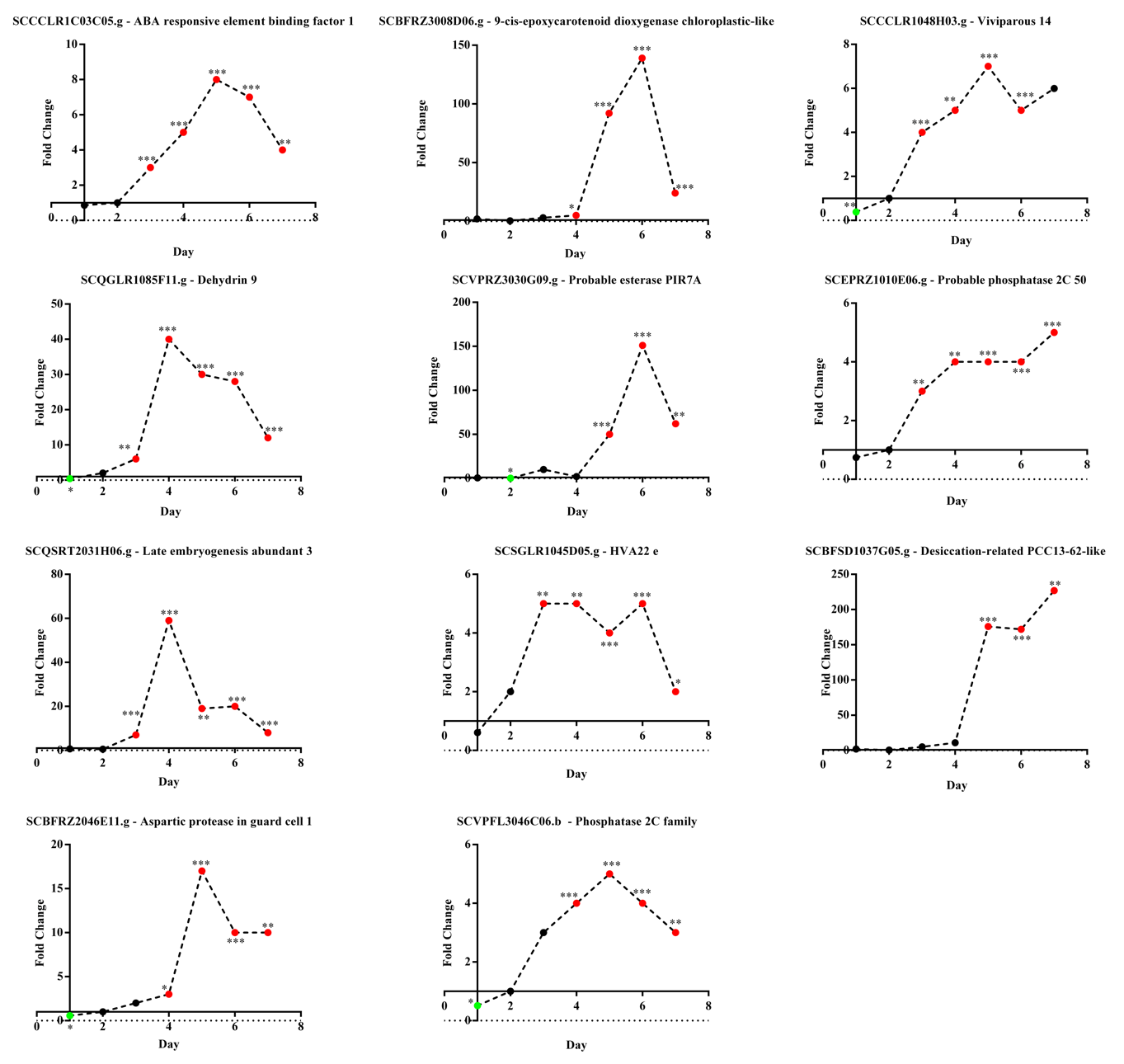


**Supplementary Figure 7 – Expression of genes related to ABA is mainly induced after 4 days of drought.**


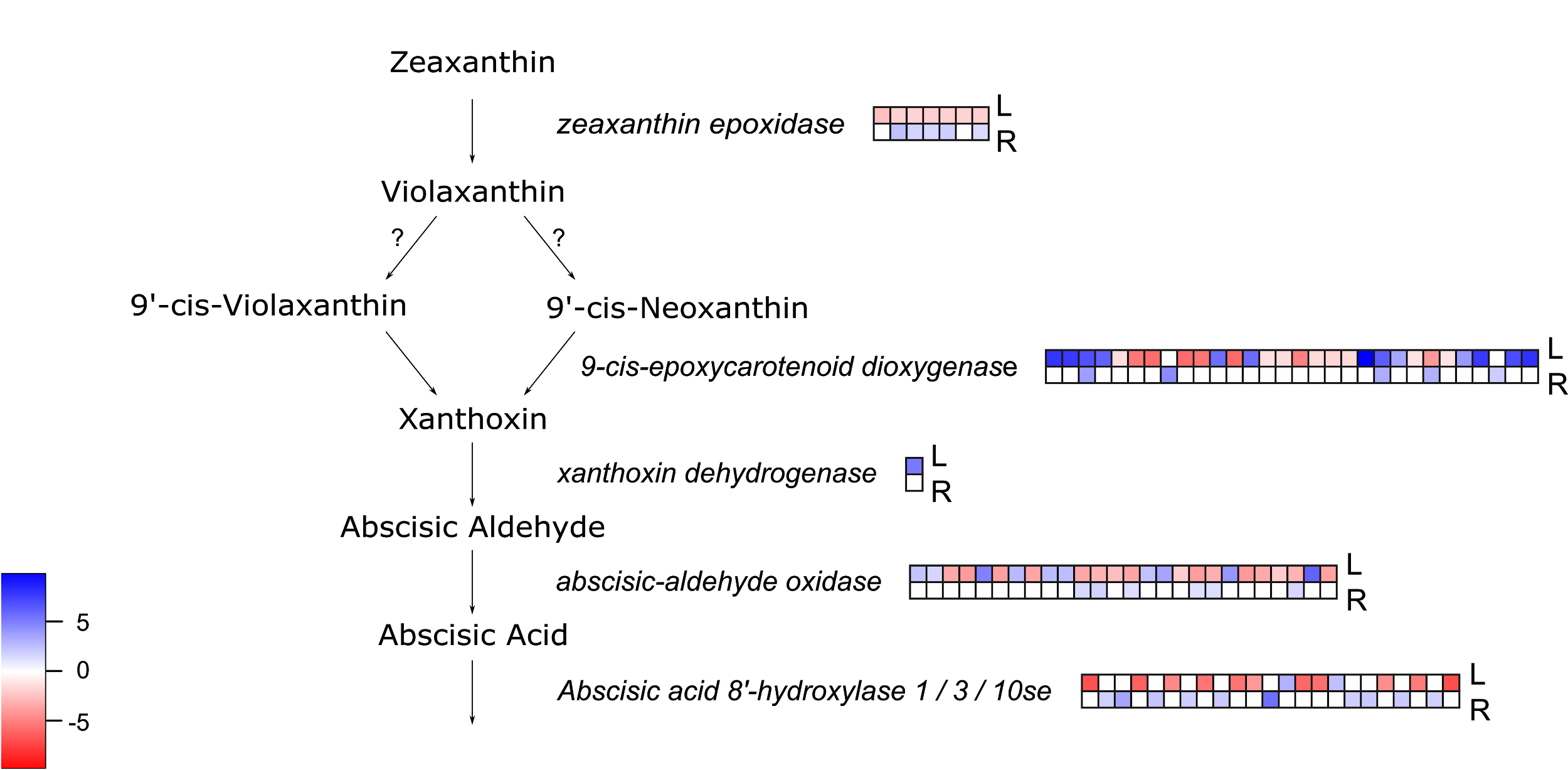


**Supplementary Figure 8 – Repression of the ABA biosynthetic pathway in leaves, and induction in roots, after 6 days of drought stress**. Each square represents expression of one transcript from the RNA-seq data after 6 days of drought. L refers to transcripts from leaf tissue, and R to transcripts from root tissue. Blue indicates induction and red indicates repression.

**A B**


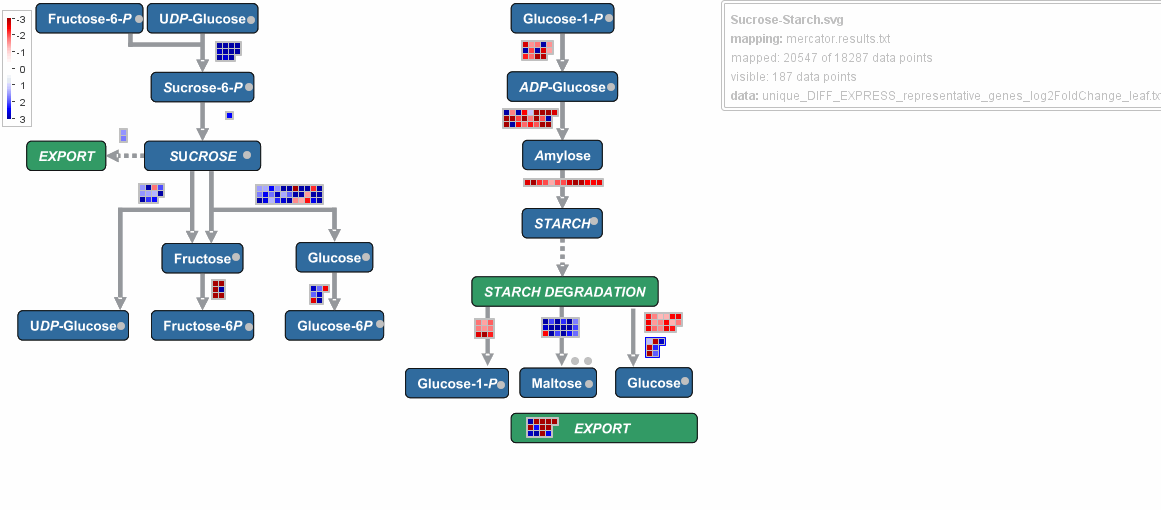


**Supplementary Figure 9 – Expression of transcripts related to sucrose synthesis (A) is induced, while starch metabolism (B) transcripts are repressed.** Mapman figures show repressed (red squares) and induced transcripts (blue squares) in RNA-seq data from leaves submitted to drought for 6 days.


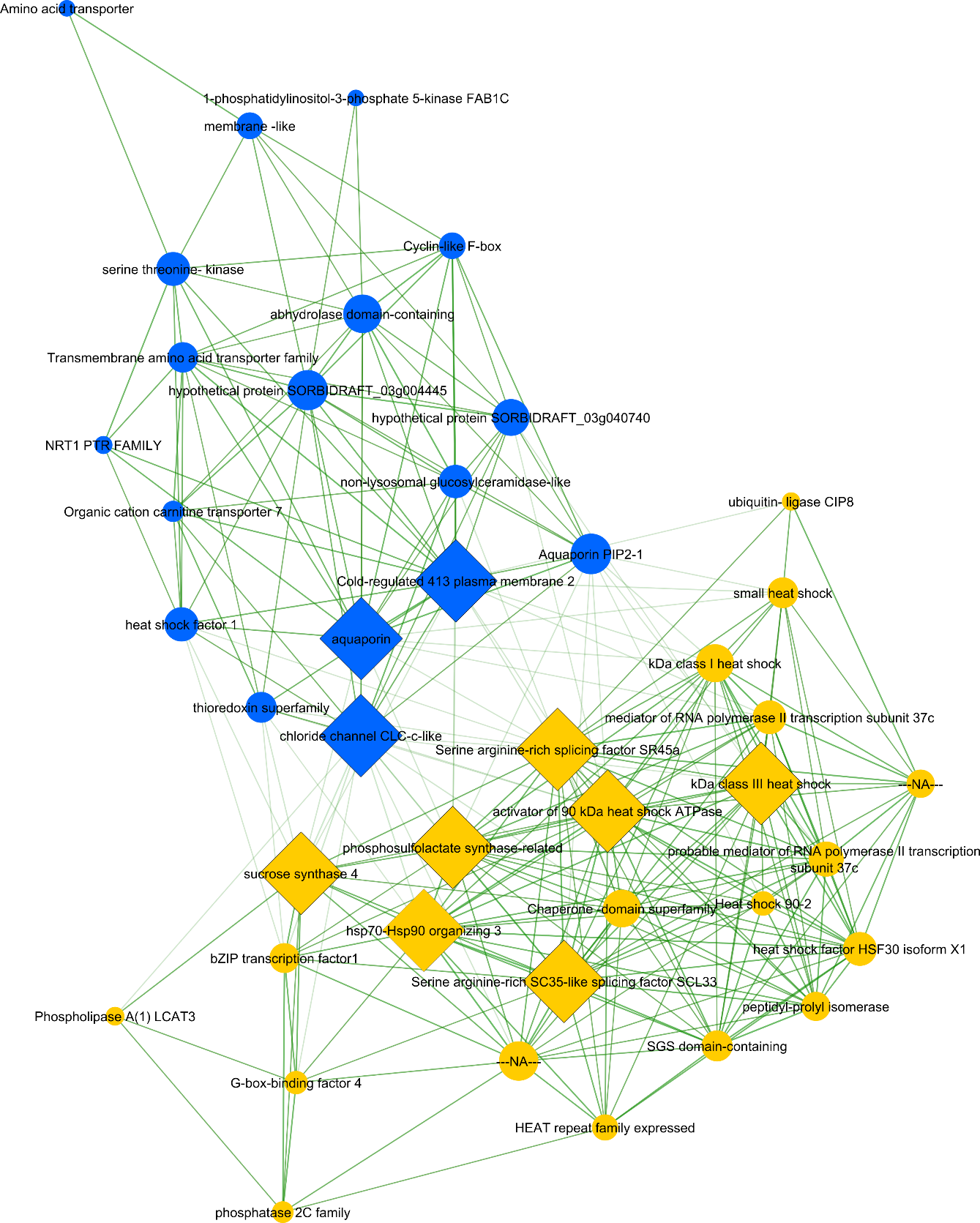


**Supplementary Figure 10 - M6 co-expression network obtained from the transcriptome of sugarcane submitted to drought.** Transcriptome data was analyzed using the CaneRegNet oligoarray platform. Diamonds indicate highly connected genes in the network (top nodes).


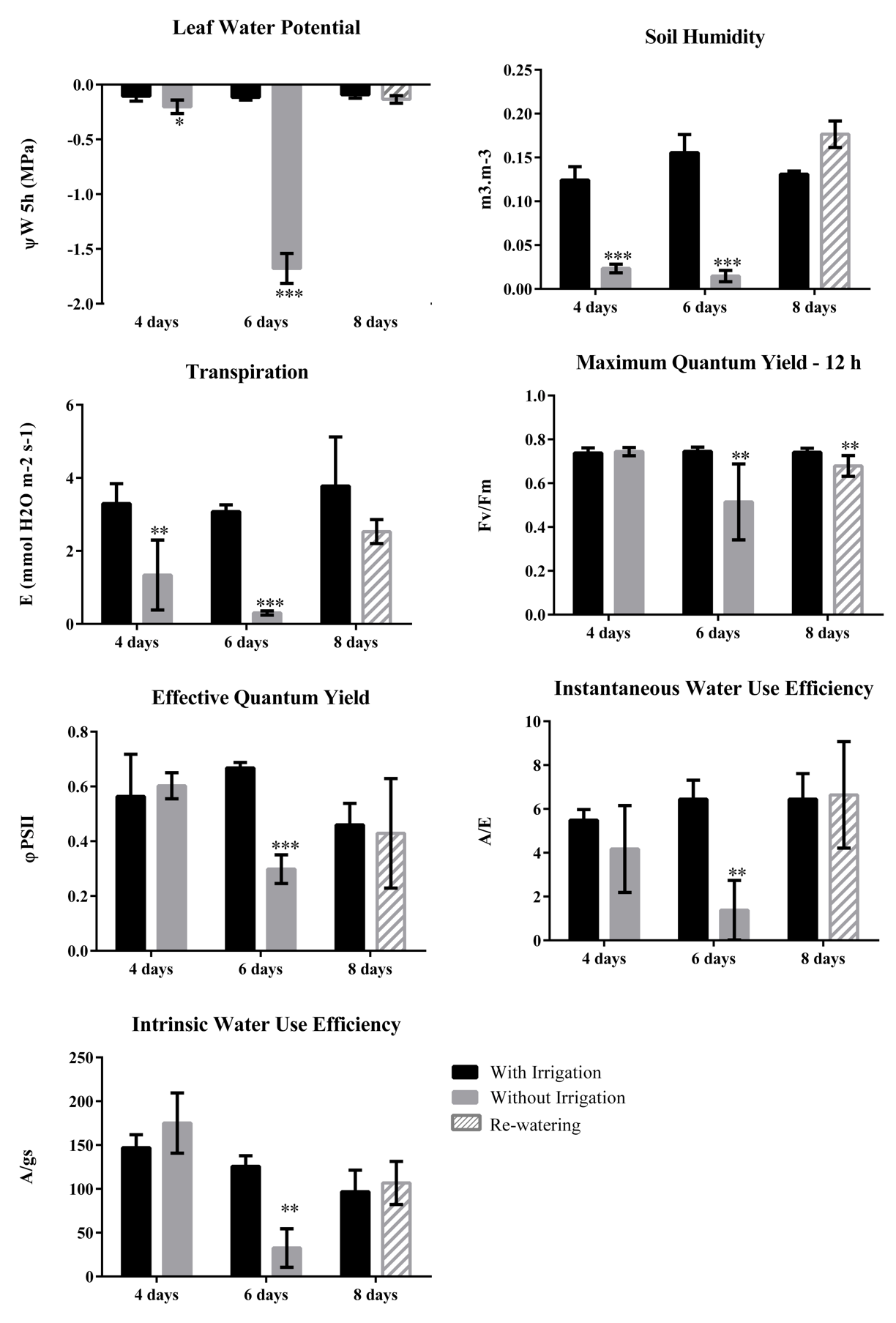


**Supplementary Figure 11 - Physiological measurements of sugarcane plants submitted to 4 and 6 days of water privation, and re-watering for 2 days after day 6 (8 days)**. Asterisks indicate significant statistical differences (t test): *, **, and *** indicate p≤0.05; p≤0.01, and p≤0.001, respectively.

**A B**


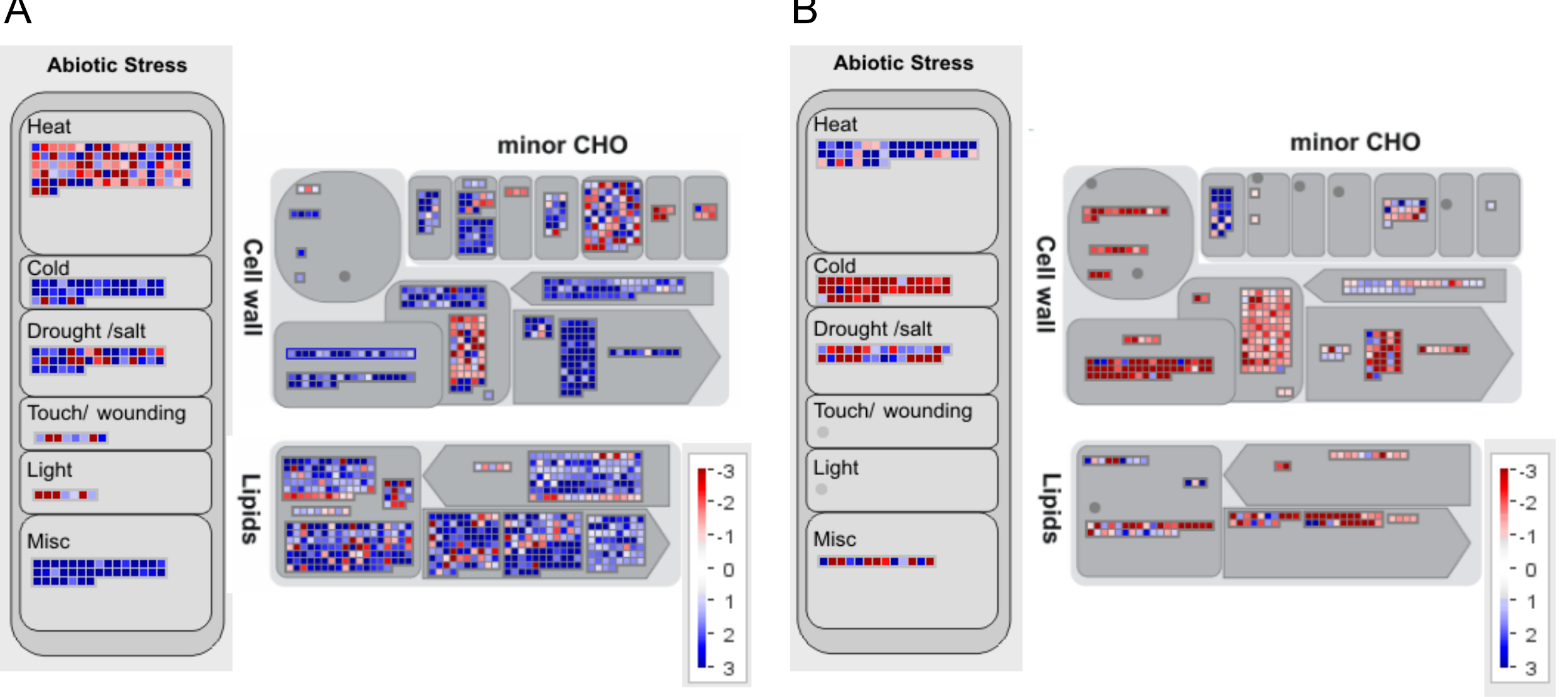


**Supplementary Figure 12 – Expression of transcripts related to abiotic stress, cell wall, and lipid metabolism are generally induced in leaves (A) and repressed in roots (B).** Transcripts from the pentose phosphate pathway are induced. Mapman figures show repressed (red squares) and induced transcripts (blue squares) in RNA-seq data from leaves and roots submitted to drought for 6 days.

**
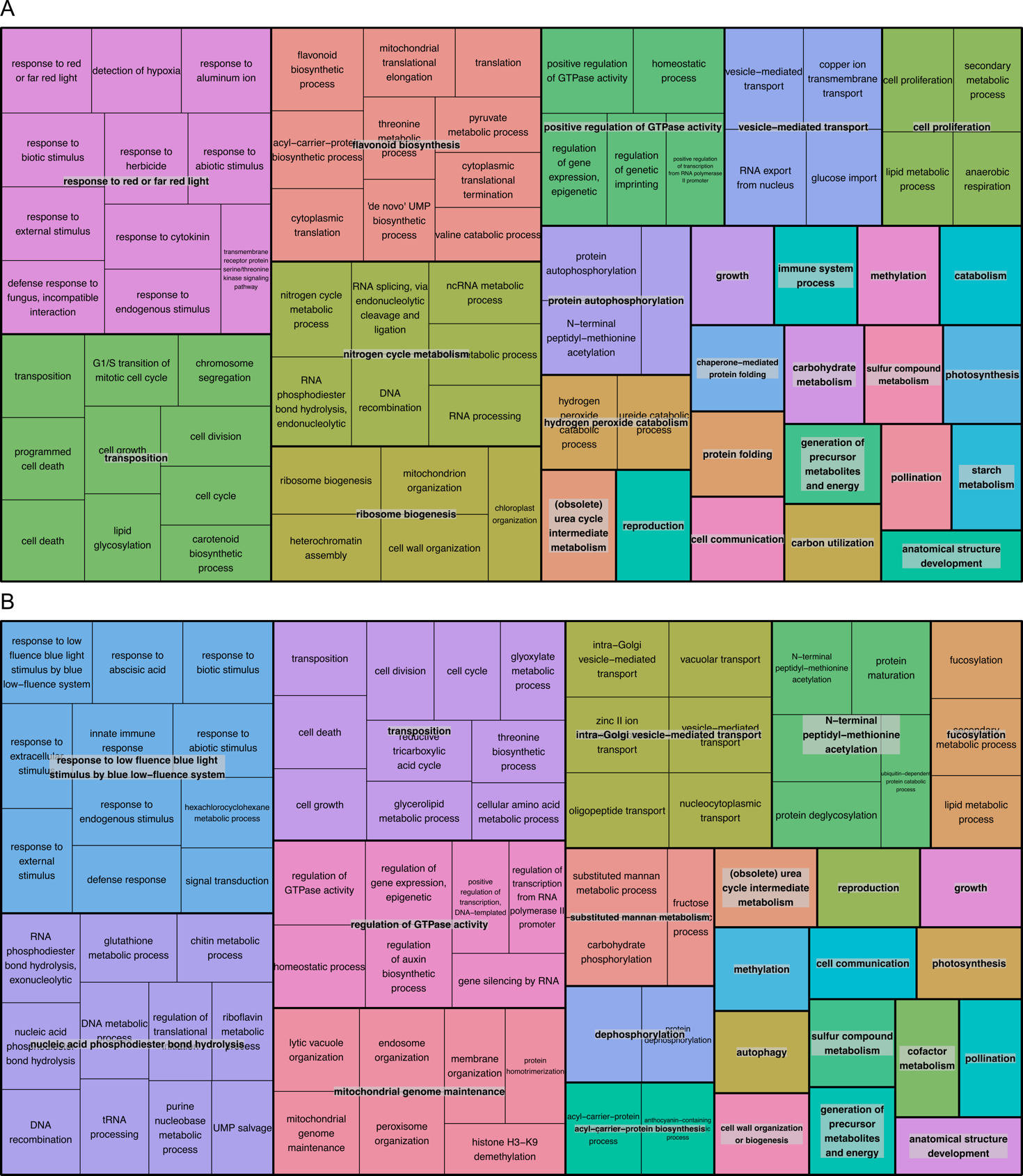
**

**Supplementary Figure 13 – Cluster analysis (REViGO) of genes responsive to drought.** Transcripts with log fold change of at least 2 (A: up-regulated) and -2 (B: down-regulated) in the oligoarray experiments and coefficient of variation higher than 120 were clustered. Highly overrepresented biological process categories are shown.


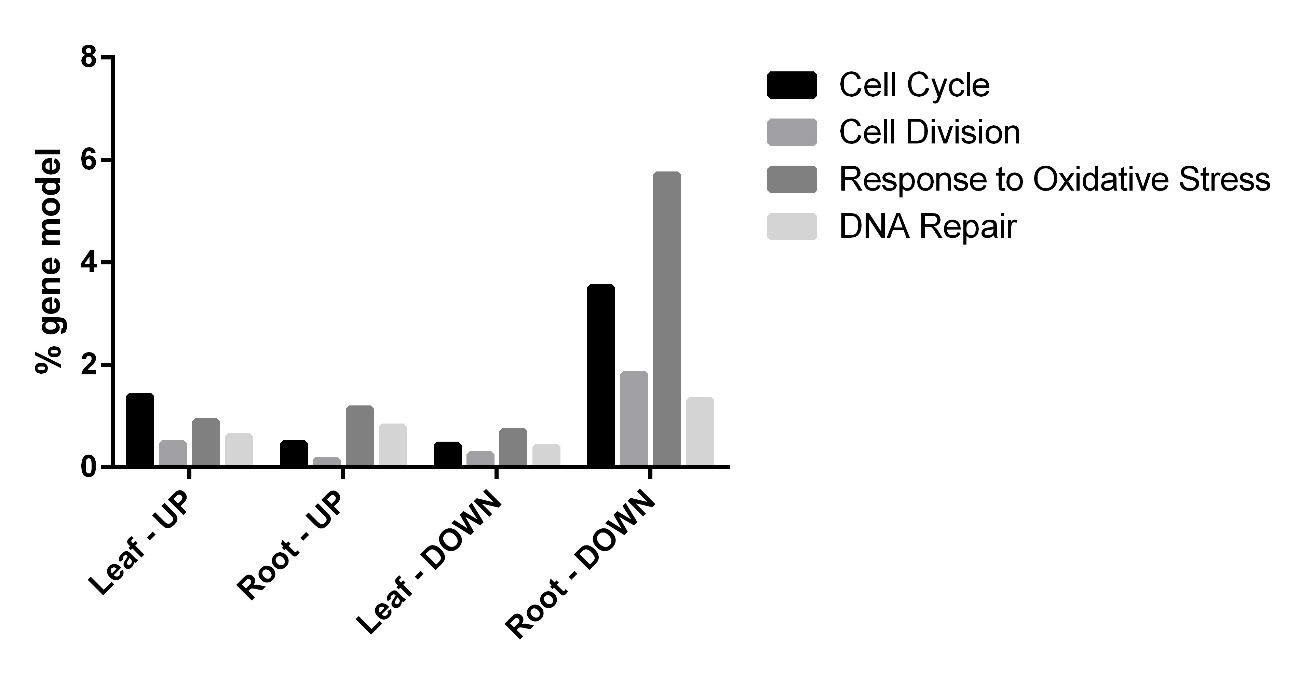


**Supplementary Figure 14 – Percentage of differentially expressed genes involved in cell cycle, cell division, redox, and DNA repair during re-watering after drought.** More genes in these categories were repressed in roots after drought stress.


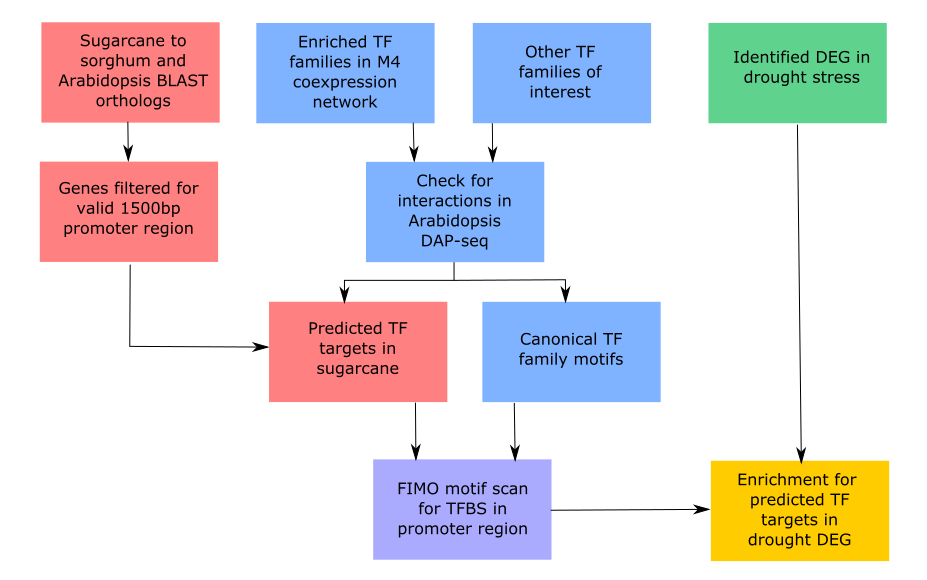


**Supplementary Figure 15 – TFBS enrichment analysis workflow.**
